# Phylogenetic Inference of H3N2 Canine Influenza A Outbreak in Ontario, Canada in 2018

**DOI:** 10.1038/s41598-020-63278-z

**Published:** 2020-04-14

**Authors:** Wanhong Xu, J. Scott Weese, Davor Ojkic, Oliver Lung, Katherine Handel, Yohannes Berhane

**Affiliations:** 1 National Centre for Foreign Animal Disease, 1015 Arlington Street, Winnipeg, Manitoba R3E 3M4 Canada; 2 Department of Pathobiology, Ontario Veterinary College, University of Guelph, Guelph, Ontario Canada; 3 Animal Health Laboratory, University of Guelph, Guelph, Ontario Canada; 4 Department of Animal Science, University of Manitoba, Winnipeg, Manitoba Canada

**Keywords:** Influenza virus, Influenza virus

## Abstract

The first Canadian H3N2 canine influenza A outbreak involving an Asian-origin H3N2 canine influenza virus (CIV) began in southwestern Ontario, Canada, in late December 2017. More H3N2 CIV cases were identified in central and eastern Ontario between March and October 2018. Based on epidemiological investigation, 5 clusters were identified (C1, C2, C3a, C3b, and C4); however, the origin of infection has only been revealed for epidemiological cluster C1. Here, we use phylogenetic analyses to unravel the links of virus transmission between the 5 epidemiological clusters and the origin of infection for all epidemiological clusters. Our results demonstrate that the Canadian H3N2 CIV sequences were grouped into four distinct phylogenetic clusters with minimal genetic diversity between these clusters. Large scale phylogenetic analysis of H3N2 CIV from around the globe showed that the Canadian CIVs formed a distinct new clade along with CIVs that have been circulating in the USA since 2017–2018 and in China since 2017. This clade shares a common ancestor of Asian origin. This study concludes that the H3N2 CIV outbreak in Ontario was driven by multiple introductions of South Korean/Chinese-origin H3N2 CIVs over 10 months.

## Introduction

Avian-origin canine influenza virus (CIV) H3N2 emerged in dogs in China and South Korea around 2005^1–3^. Since then, H3N2 CIVs have repeatedly been isolated in dogs in both China and South Korea and the geographical distribution of this virus has been rapidly expanding. The H3N2 CIV was isolated from dogs in Thailand in 2012^4^ and reached the United States in early 2015 through the importation of dogs from South Korea and China^5^. This virus has since caused an ongoing epidemic of disease through multiple, periodic introductions of Asian-origin H3N2 CIVs in the United States^5,6^.

**Figure 1:**
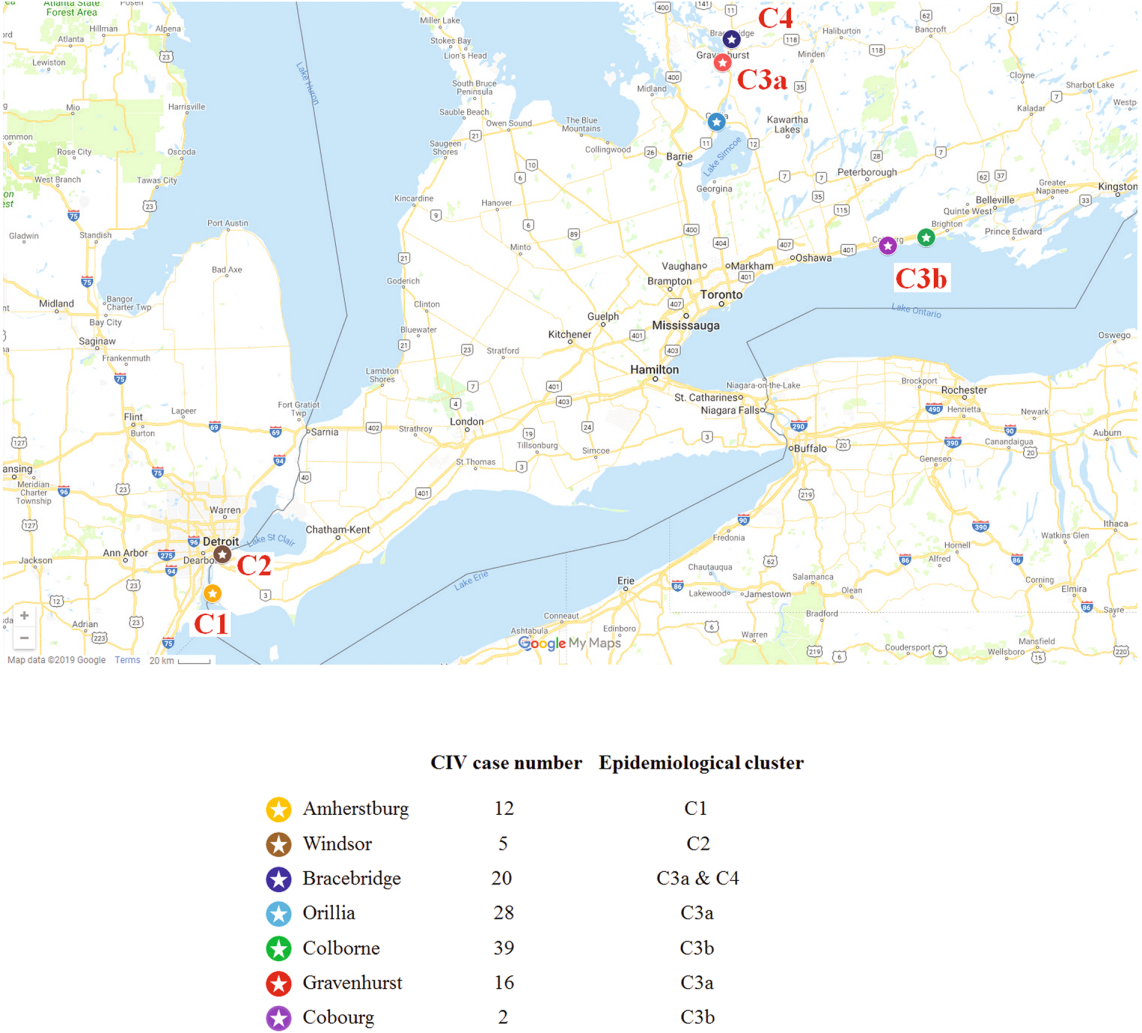
Map indicating the locations of H3N2 cases identified during the 2018 outbreak in Ontario, Canada. Cities are represented by colored stars, according to their location and inclusion in an epidemiological cluster of infection. The satellite figure map was produced from Google Maps (Map data©2019 Google; https://www.google.com/maps/place/Canada) complying with the Terms of Service as outlined at https://www.google.ca/permissions/geoguidelines.html with modifications.

H3N2 CIV had not been identified in Canada until the end of 2017^7^. Multiple introductions with subsequent transmission of H3N2 CIV were identified in the Canadian province of Ontario between December 28, 2017, and October 30, 2018. Five epidemiological clusters (C1, C2, C3a, C3b, and C4) of the H3N2 CIV infection in Ontario were defined with various numbers of CIV cases identified in Amherstburg, Windsor, Bracebridge, Orillia, Colborne, Gravenhurst, and Cobourg (Fig. 1). The origin of cluster C1 was associated with the importation of H3N2-infected dogs from South Korea^7^; the origin of cluster C2 was unclear, and the origin of clusters 3 and 4 was presumably linked to dogs imported from China. Intensive contact tracing, testing of contacts and a 28-day isolation period for both infected and exposed dogs were implemented throughout the outbreak.

Phylogenetic studies have largely contributed to a better understanding of the emergence, spread and evolution of many RNA viruses, such as the foot-and-mouth disease outbreak in the United Kingdom in 2001^8^, highly pathogenic avian influenza epidemics^9–11^, and 2013**–**2015 Ebola virus epidemic in West Africa^12,13^. To gain a better understanding of H3N2 CIV emergence in Ontario and the evolutionary dynamics of this outbreak, 21 samples from five epidemiological clusters were randomly selected for full genome sequencing. Both Maximum Likelihood (ML) and Bayesian approaches were used to infer phylogenetic relatedness. Here we provide an analysis of the complete coding-region of eight gene segments of 21 H3N2 CIV strains that were sampled from infected dogs in Ontario, Canada in 2018. We compared their full genomes to 162 H3N2 CIV full genomes that were available in the GenBank database. We used this combined data set to better understand the molecular evolution of H3N2 CIV, including the nucleotide substitution rate and selection pressure.

## Results

### Sequence analysis

Strains from epidemiological clusters C1 and C2 showed complete coding lengths for all eight gene segments (i.e. PB2, 759 aa; PB1, 757 aa; PB1-F2, 90 aa; PA, 718 aa; PA-X, 232 aa; HA, 566 aa; NP, 498 aa; NA, 469 aa; M1, 252 aa; M2, 97 aa; NS1, 230 aa; NS2, 121 aa). Strains from epidemiological clusters C3a, C3b, and C4 showed complete coding lengths for seven out of eight gene segments (i.e. segments 1 to 7). The segment 8 encodes nonstructural proteins NS1and NS2. The NS1coded for 217 aa due to a premature stop codon at position 218 for strains of epidemiological clusters C3a, C3b, and C4. Pairwise sequence analysis of 21 Canadian H3N2 strains showed a nucleotide identity of 98.9**–**100% and an amino acid identity of 98.1**–**100% among the segments 1 to 7. A comparison of nucleotide substitutions with the first H3N2 CIV isolate Guangdong/1/2006 revealed 52**–**65 nucleotide substitutions in PB2 in the 21 Canadian strains (nonsynonymous substitutions:16**–**17), 59**–**81 for the PB1 gene segment (nonsynonymous substitution: 12**–**16), 57**–**80 for the PA gene segment (nonsynonymous substitution: 15**–**18), 41**–**66 for H3 (nonsynonymous substitution: 14**–**16), 37**–**50 for NP (nonsynonymous substitution: 7**–**8), 41**–**58 for N2 (nonsynonymous substitution: 15**–**19), 19**–**25 for the M gene segment (nonsynonymous substitution: 12**–**13), and 26**–**41 for the NS gene segment (nonsynonymous substitution: 7**–**12). Interestingly, out of twenty-seven amino acid mutations that differentiate the current H3N2 CIV isolates from other avian influenza viruses (AIVs) reported in a previous study^14^, twenty of these amino acids remained unchanged in this study (Table 1), suggesting these amino acids were important for dog adaptation.

**Table 1:** Amino acids that differentiate H3N2 CIV from AIVs present in all H3N2 CIVs in this study. ^a^Codon position. H3 numbering is used for codon positions in HA.

Segment	Position^a^	AIV residue	H3N2 CIV residue
PB2	147	I	T
	570	M	V
PB1	108	L	I
	361	S	N
PA	369	A	V
	615	K	R
HA	81	D	N
	222	W	L
	489	D	N
NA	54	E	K
	81	P	S
	143	D	N
	156	P	S
	372	S	L
	432	R	G
NS1	60	A	I
	67	R	W
	75	E	K
	152	E	N
	172	E	K

No evolutionary divergence was observed within the five epidemiological clusters at either the nucleotide or amino acid levels. The nucleotide and amino acid differences found in the concatenated eight gene segments of the current study’s 21 Canadian strains are summarised in Table 2. No divergence was observed between epidemiological clusters C1 and C2 at either the nucleotide or amino acid levels, suggesting the source of infection for cluster C2 was the same as for cluster C1.

**Table 2:** The evolutionary divergence over sequence pairs between epidemiological clusters. Percent of amino acid differences indicated in the upper triangle and percent nucleotide differences in the lower triangle.

Cluster	C1	C2	C3a	C3b	C4
C1		0.00	0.50	0.46	0.59
C2	0.00		0.52	0.48	0.61
C3a	0.52	0.53		0.04	0.55
C3b	0.49	0.50	0.04		0.50
C4	0.61	0.62	0.57	0.57	

### Phylogenetic analysis

**Figure 2:**
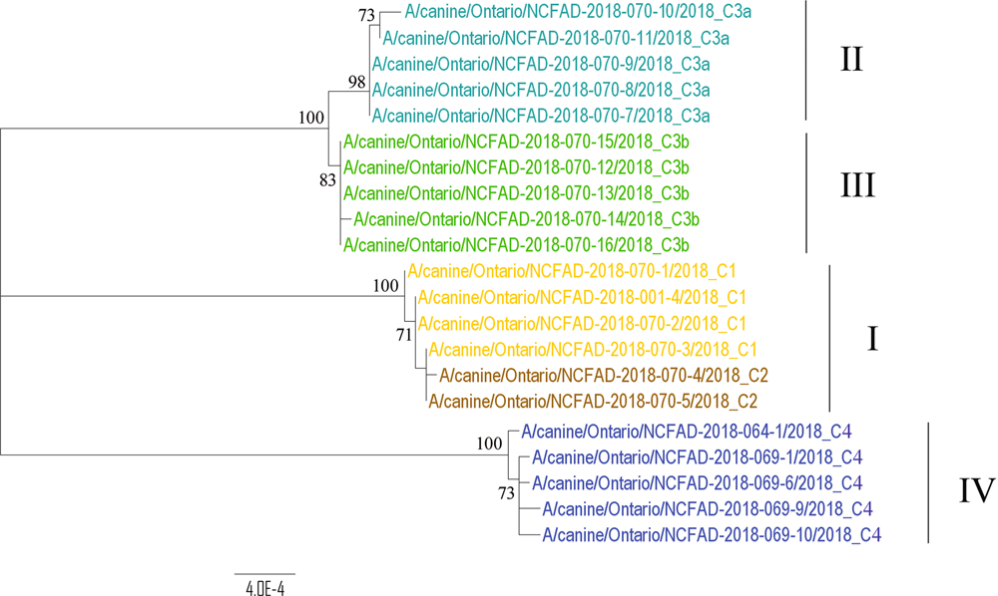
Maximum Likelihood tree of the concatenated eight gene segments of Canadian H3N2 CIVs. Viruses are colored by their epidemiological clusters (yellow for cluster C1; brown for cluster C2; cyan for cluster C3a; green for cluster C3b; and blue for cluster C4). Phylogenetic clusters are denoted as I to IV. The numbers at nodes represent bootstrap values (>70%), while branch lengths are scaled according to the numbers of nucleotide substitutions per site. The tree is midpoint rooted for clarity.

**Figure 3:**
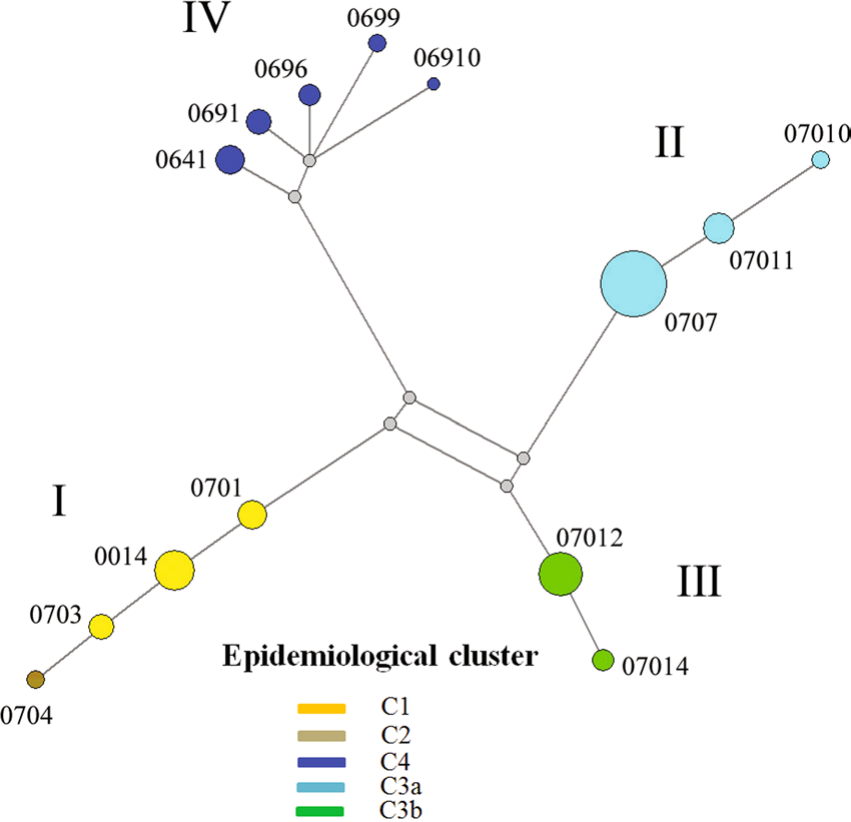
Median-joining phylogenetic network of Canadian H3N2 CIVs. The median-joining network was constructed from the concatenated eight gene segments. This network includes all of the most parsimonious trees linking the sequences. Each unique sequence is represented by a circle whose size reflects the frequency of the sequence in the data set. The branch length is proportional to the number of mutations. Strains are colored according to their epidemiological clusters.

The origin of H3N2 CIV infection in southwestern Ontario that was clearly defined by epidemiologic investigation and molecular testing was cluster C1 (South Korean origin). The origins of H3N2 CIV infection in central Ontario and eastern Ontario that were probably linked to different imported dogs from China were clusters C3a, C3b, and C4. The origin of H3N2 CIV infection in cluster C2 was suggested to link to cluster C1 due to the temporal and geographic proximity and the lack of any known contact with imported dogs. Phylogenetic trees of the 21 H3N2 CIVs that were sampled from five epidemiological clusters were constructed for the eight separate gene sequence datasets using the Maximum Likelihood method (Supplementary Fig. S1). Both Bayesian inference and Maximum Likelihood methods were used for concatenated gene sequences of 21 H3N2 CIVs (Supplementary Fig. S2, Fig. 2). In general, the topology of phylogeny from each gene segment showed similar clustering patterns as the concatenated gene segments (Fig. 2), i.e., gene sequences grouped corresponding to their geographic locations. Four phylogenetic clusters were identified: (i) Cluster I contained strains from epidemiological clusters C1 (Amherstburg) and C2 (Windsor), in which samples were collected in January and early February of 2018; (ii) Cluster II contained strains from epidemiological cluster C3a (Orillia and Bracebridge), in which samples were collected in early March of 2018; (iii) Cluster III contained strains from epidemiological cluster C3b (Colborne), in which samples were collected in middle and late March of 2018; (iiii) Cluster IV contained strains from epidemiological cluster C4 (Bracebridge), in which samples were collected in October of 2018. This cluster was assumed to have resulted from a separate importation of dogs from China, months after the previous introduction of CIV had been eliminated, as all affected dogs had contact with a facility that imported dogs that month. Epidemiological cluster C3a was suspected to have had the same disease origin as cluster C3b, as a dog from the group imported from China to the index facility for cluster C3a was moved to a different part of Ontario (Niagara region), with subsequent transmission to other dogs. The dog that was identified as the index case in cluster C3b had been obtained from an animal shelter in the Niagara region, with subsequent transmission of CIV to dogs in the region where cluster C3b occurred. Unfortunately, based on the timing of the investigation and testing, adequate samples were not available from the Niagara region cases. To assess the inter-city transmission network, the single alignment from the concatenated eight gene segments was used to construct a Median Joining (MJ) phylogenetic network (http://www.fluxus-engineering.com) (Fig. 3). The network obtained included all the most parsimonious trees, thus representing all the plausible evolutionary pathways linking the outbreak samples. The network showed that the virus sequences were grouped into four clusters of infection that were identified in the ML and MCC trees. Sequences within these 4 clusters were separated by 1**–**3 nucleotide differences, whereas 72**–**86 differences were observed between clusters except between clusters II and III where 6 nucleotide differences were observed. Both ML/ Bayesian and MJ phylogenetic analyses indicated that epidemiological cluster C2 had the same disease origin as cluster C1. This suggests that the infection of epidemiological cluster C2 was due to contact with infected dogs from cluster C1.

**Figure 4:**
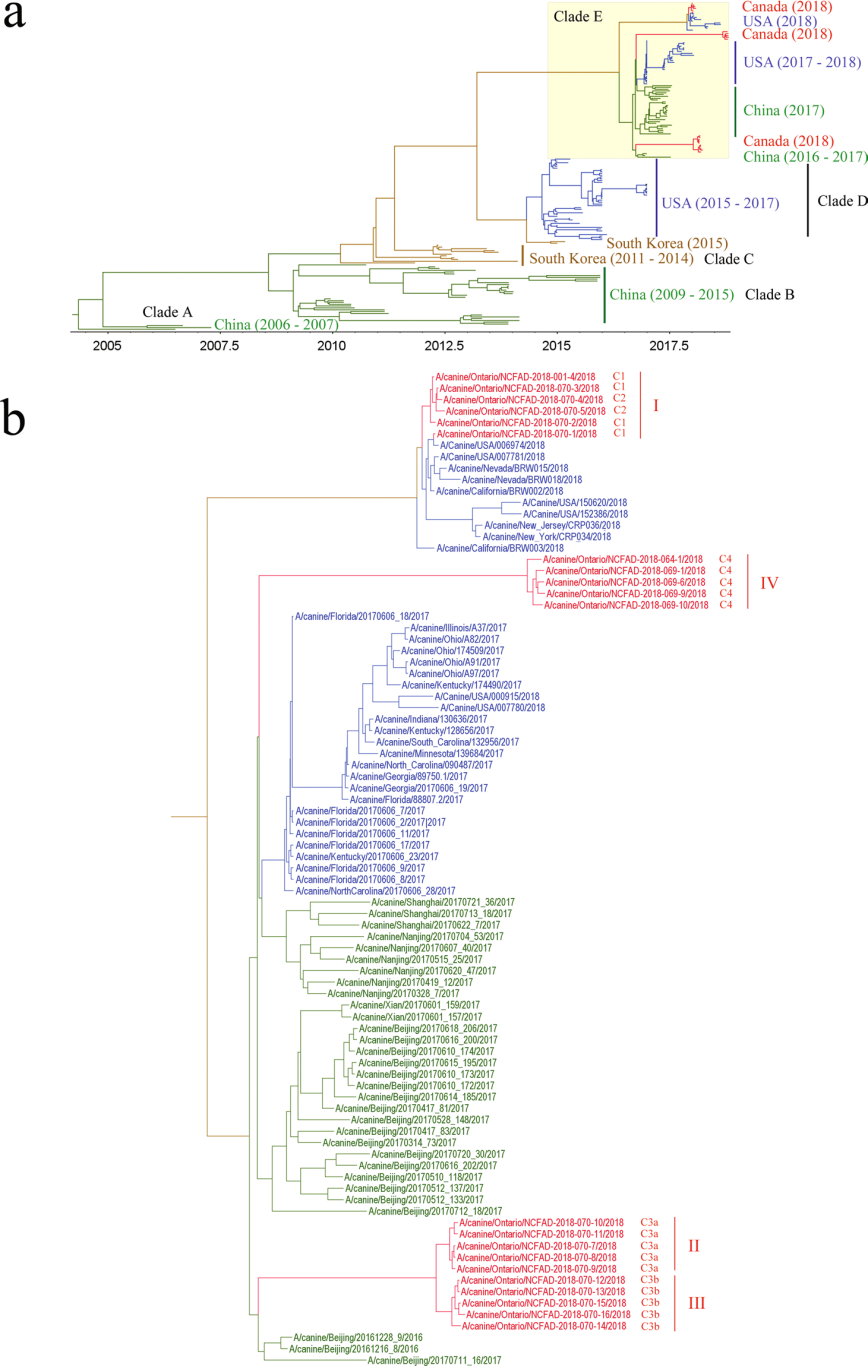
(**a**) Maximum clade credibility tree inferred for the concatenated eight gene segments of 183 H3N2 CIVs. Sequences are colored according to the country of origin. Red, Canada; blue, the United States; green, China; brown, South Korea. Global clades are denoted as A to E. (**b**) Close-up of clade E from A with the name of viruses labeled. Epidemiological and phylogenetic clusters defined for Canadian H3N2 CIV outbreak strains are indicated.

To explore the phylogenetic relationships of the H3N2 CIVs on a global scale, MCC trees were inferred for individual genomic segments for a total of 183 H3N2 CIV strains collected from 2006 to 2018. Of these strains, 21 were from Canada, 81 from the USA, 68 from China, and 13 from South Korea. Phylogenies inferred for each of the eight genome segments were shown in Supplementary Fig. S3. An MCC tree generated from the concatenated eight gene segments of all 183 strains were shown in Fig. 4a. Five distinct clades were identified (i.e. clades A, B, C, D, and E). The topology of the concatenated gene phylogenies demonstrated that the H3N2 CIVs clustered together by country (i.e. China and South Korea) as monophyletic groups before the year of 1995. The topology of phylogenies has changed to include the strains from the United States starting in 1995 and strains from Canada in 2018 (Fig. 4a). The Canadian outbreak viruses located in the global clade E were the same distinct cluster structure as seen in Fig. 2 (Fig. 4b).

### Evolutionary rate and origin

The rates of nucleotide substitution and the times to the most recent common ancestor (tMRCA) of the 183 H3N2 CIV strains were estimated for each genome segment separately and for the eight concatenated segments using a BMCMC method in BEAST^15,16^. The means and 95% highest posterior density (HPD) intervals of all genomic segments are shown in Table 3. There were no significant differences in nucleotide substitution rates among any genome segments of H3N2 CIVs. Previous studies have found higher nucleotide substitution rates for the genes encoding the surface glycoproteins in highly pathogenic avian influenza viruses^11,17,18^. However, the nucleotide substitution rate of 1.34 × 10^**−**3^ subs/site/year from the concatenated gene segments during 12 years of evolution was similar to that reported by Voorhees *et al*.^6^. The mean of tMRCAs for each of the eight gene segments ranged from 1995 to 2005 (Tables 3), 1 to 11 years before the first isolation of H3N2 CIV in 2006 in China.

**Table 3:** Mean nucleotide substitution rates and estimation of time of the most recent common ancestor for each gene segment of H3N2 CIVs. ^a^Concatenated gene segments.

Gene	Substitution rate (subs/site/year)		tMRCA (yr)	
	Mean (X 10^**−**3^)	95% HPD (X 10^**−**3^)	Mean	95% HPD
PB2	1.57	1.33**–**1.85	2004	2002**–**2005
PB1	1.58	1.36**–**1.80	2003	2002**–**2004
PA	1.42	1.20**–**1.62	2004	2003**–**2005
HA	2.1	1.77**–**2.42	2004	2003**–**2005
NP	1.59	1.29**–**1.88	2002	2000**–**2004
NA	1.64	1.38**–**1.92	2005	2003**–**2006
M	1.49	1.19**–**1.79	2004	2003**–**2006
NS	2.13	1.72**–**2.57	1995	1990**–**1998
CG^a^	1.34	1.23**–**1.47	2003	2002**–**2004

### Selection pressures acting on the viral genes

An analysis of selection pressures on all of the viral genes revealed that the vast majority of codons were subject to purifying selection. Using the SLAC, FEL, FUBAR, and MEME methods, we identified several sites in PB2, PB1, PA, HA, and NA with evidence of putative positive selections (Table 4), while no positive selections were found under the FEL method. Amino acids under positive selection in HA and NA genes were found within experimentally determined epitopes^19^, while the phenotypic consequence of the positive selections identified in PB2, PB1, and PA genes are unknown.

**Table 4:** Amino acid sites under putative positive selection detected using different analytical models. ^a^Codon position. H3 numbering for HA is in brackets.

Gene	Codon^a^	SLAC		FUBAR		MEME	
		dN-dS	p-value	dN-dS	Posterior probability	p-value	Number of branches under episodic selection
PB2	35					0.000	1
PB1	37					0.000	1
	143	11.41	0.088				
	295					0.000	1
PA	300					0.010	1
HA	47 (31)					0.010	1
	351 (335)					0.000	3
	380 (364)					0.000	
	469	14.33	0.098	6.711	0.921		
NA	49					0.000	
	60					0.000	
	340					0.000	

## Discussion

Our findings from 21 H3N2 CIV genomes sampled in Ontario over 10 months during the 2018 H3N2 CIV outbreak in Canada demonstrate the value of phylogenetic analysis. We identified 4 phylogenetic clusters within the Canadian CIV outbreak using both the Maximum Likelihood and Bayesian methods. As clusters II and III were more closely related, this suggests that at least three introductions of CIV H3N2 occurred during the Canadian outbreak. While the origin and source of infection of epidemiological cluster I was correctly determined by a previous epidemiological investigation and molecular detection^7^, the origins of infection of clusters II, III, and IV remained unanswered. In this study, we could show that clusters II and III shared a common ancestor of Chinese origin in the global phylogenetic tree (Fig. 4b), despite not having molecular testing data from imported Chinese dogs during the outbreak (7). Considering the time of infection, clusters II and III viruses were all sampled in March of 2018. Clusters II and III viruses might have resulted from a single introduction but a virus strain of a different genetic background. Global phylogenetic analysis of H3N2CIV showed that the Canadian H3N2 CIV outbreak strains belong to clade E (Fig. 4a). This clade is composed of recent Chinese H3N2 CIVs, the U.S. H3N2 CIVs, and Canadian H3N2 CIV outbreak strains with a common ancestor being of Asian origin. Phylogenetic analyses in this study supported the origin and source of H3N2 CIV infection in Ontario that was inferred by the epidemiologic analysis.

The sequence analysis of 21 Canadian H3N2 viruses revealed that these newly emerged viruses were all very similar with over 98% identity at both nucleotide and amino acid levels for all eight gene segments. We also did not see any evolutionary divergence within the five epidemiological clusters. The nucleotide substitution rate of the concatenated genome for the 21 Canadian H3N2 CIVs was 2.9 × 10^**−**3^ subs/site/year while 1.34 × 10^**−**3^ subs/site/year were observed at a global level for 12 years. The evolutionary dynamics of Canadian H3N2 CIV outbreak strains exhibited in this study are attributed to the fact that Canadian H3N2 CIVs were the result of multiple introductions of South Korean/Chinese-origin H3N2 CIV. Each introduction was contained within a month so that the viruses did not have sufficient time to diverge into more naïve dog populations within Ontario or spread to other provinces within Canada. Meyer *et al*. have reported that at least 3**–**4 months of temporal divergence from the start of sampling was required to make a precise estimate that agreed with a long-term value for nucleotide substitution rate^20^.

We showed a low nucleotide substitution rate of 1.34 × 10^**−**3^ (subs/site/year) in the 12 years’ evolution period of H3N2 CIV, which is consistent with reports by other researchers^6,14,21^. Compared to the first H3N2 CIV isolate Guangdong/1/2006 from China, the Asian-origin Canadian H3N2 CIVs have displayed numerous non-synonymous mutations in all 8 gene segments despite not having evolutionary divergence within clusters and minimum divergence between clusters observed. Selection pressures acting on 183 H3N2 CIV genomes collected globally revealed that the vast majority of codons underwent purifying selection with 12 codons located in PB2, PB1, PA, HA, and NA having been subjected to putative positive selection. We did not find any evidence that these positive selection sites were associated with phenotypic consequences. Further experimental work is needed to demonstrate if the amino acid changes described in the current study contribute to any phenotypic changes that might increase or alter the virulence of the virus.

In summary, the outbreak in Ontario was driven by at least three separate introductions of Asian-origin H3N2 CIVs from imported dogs which originated from countries that are known to have endemically infected canine populations. The H3N2 CIV continues to evolve at a low mutation rate and geographic clustering features entire phylogenies. It is suggested that imported dogs from endemic countries should be quarantined and monitored before releasing them to pet owners. This can prevent transmission and spread of CIV into immune naïve dog populations.

## Methods

### Viral sequences

Twenty-one samples sequenced in this study were obtained from the Animal Health Laboratory, University of Guelph, Ontario, Canada. All sample collections were conducted at Ontario Veterinary College in the course of routine treatment of the dogs according to the procedures approved by the University of Guelph Animal Care Committee in accordance with the Canadian Council on Animal Care Guidelines. Samples were collected in virus transport medium and stored at **−**70 °C freezer. Viral RNA was extracted using the MagMaxTM RNA isolation kit (Life Technologies, Carlsbad, CA, USA), and the entire genome (PB2, PB1, PA, HA, NP, NA, M, and NS) of each virus was amplified using Uni12 and Uni13 primers as described previously^22^. RT-PCR was performed using the qScript XLT one-step RT-PCR kit (Quanta Biosciences Inc). PCR run conditions consisted of 42 °C for 60 min, 94 °C for 2 min, 5 cycles of (94 °C for 30 sec, 44 °C for 30 sec, 68 °C for 3.5 min), 26 cycles of (94 °C for 30 sec, 57 °C for 30 sec, 68 °C for 3.5 min), and then 68 °C for 10 min. PCR products were purified using the QIAquick PCR purification kit (Qiagen) and the concentration of each PCR amplicon was determined with the Qubit® Fluorometer and Qubit® dsDNA BR (Broad Range) Assay Kit (Invitrogen). Two types of technologies were used for full-genome sequencing. (A) Ion Torrent PGM™ Technology. The Library Prep was done using the ABI AB Library Builder (Life Technologies) with the IonXpress™ Plus Fragment Library Kit for AB Library Builder™ and IonXpress™ Barcodes with 6 minutes of DNA shearing time. This was followed by 8 cycles of amplification with Invitrogen Platinum™ SuperMix High Fidelity Taq on a thermal cycler dedicated to library prep. Library Purification with Agencourt AMPure XP beads was performed using the published Ion Torrent protocol (ThermoFisher Publication Number MAN0007044). The DNA template concentration for Emulsion PCR was determined by qPCR with the Ion Library Quantification kit on the ABI 7500 Fast Real-Time PCR instrument. Libraries were diluted and pooled before Emulsion PCR with the Ion OneTouch2 / ES system and Ion Hi-Q™ View OT2 kit. Sequencing was done on the Ion Torrent PGM™ with Torrent Suite™ v 5.6 software using the Ion Hi-Q™ Sequencing kit and Ion 314 Chip v2 BC according to the Manufacturer’s protocol. (B) Illumina MiSeq Technology. Library prep was done with the Illumina Nextera XT Library Preparation Kit. Briefly, PCR amplicons were normalized before Tagmentation, followed by 12 cycles of amplification to add Nextera XT Index Adapters on a thermal cycler dedicated to library prep, followed by purification with Agencourt AMPure XP beads. The concentration of individual libraries was determined with the Qubit® Fluorometer and Qubit® dsDNA HS (High Sensitivity) Assay Kit (Invitrogen) and then Libraries were pooled and an aliquot was run on the Agilent 2100 Bioanalyzer with an Agilent High Sensitivity DNA kit to determine the average base pair (bp) size of the Library Pool. The concentration of the Pooled Library was re-measured with the Qubit® Fluorometer, and then the Pooled Libraries were diluted to 2 nM, denatured with 0.2 N NaOH and diluted with HT1 Buffer to a final concentration of 10 pM with a 1% spike-in of 12.5 pM PhiX v3 Control. An Illumina V2 Paired-End 300 cycle Cartridge with a MiSeq Nano Flow Cell was used to run the samples on an Illumina MiSeq. Each genome segment was assembled utilizing the DNAstar SeqMan NGen software (Version 15.3.0; DNASTAR, Inc).

### Nucleotide sequences used in the study

All available full genome sequences of H3N2 CIV were downloaded from the Influenza Research Database (https://www.fludb.org/) on May 5, 2019. Sequences containing ambiguous bases were removed after initial alignment using Muscle in MEGA-X^23^. Alignments of each genome segment were screened for recombinant sequences using the programs RDP, GENECONV, MAXCHI, CHIMAERA, 3SEQ, BOOTSCAN and SISCAN in the recombination detection program version 4 (RDP4) software package^24^ using default settings. Potential recombinant sequences were identified when two or more methods were in agreement with p-values <0.001. A total of 162 H3N2 CIV full genome sequences (Supplementary Table S1) from the Influenza Research Database were free of recombinant events when compiled with the 21 full genome sequences generated in this study (Table 5). This led to a data set consisting of 183 H3N2 CIV full genome sequences that were analyzed in this report.

**Table 5:** Canadian H3N2 CIV samples used in this study.

Strain name	ID in MJ network	Sample collection date (y/m/d)	Location	History of infected dog	GenBank accession no.	Sequence reference
NCFAD-2018–064–1	0641	2018–10–15	Bracebridge	Boarded at rescue	MN586079-MN586086	This study
NCFAD-2018–069–1	0691	2018–10–22	Bracebridge	History unknown	MN586087-MN586094	This study
NCFAD-2018–069–6	0696	2018–10–22	Bracebridge	Infected via groomer	MN586103-MN586110	This study
NCFAD-2018–069–9	0699	2018-10-24	Bracebridge	Boarded at rescue	MN586111-MN586118	This study
NCFAD-2018-069-10	06910	2018-10-18	Bracebridge	Contact with imported dog from rescue	MN586095-MN586102	This study
NCFAD-2018-001-4	0014	2018-01-05	Amherstburg	Contact with infected dog	MN586071-MN586078	This study
NCFAD-2018-070-1	0701	2018-01-09	Amherstburg	Contact with the imported dog from South Korea	MN586119-MN586126	This study
NCFAD-2018-070-2	0702	2018-01-09	Amherstburg	Exposed to the dog that had contact with the imported dog	MN586183-MN586190	This study
NCFAD-2018-070-3	0703	2018-01-13	Amherstburg	Foster household, contact with the imported dog	MN586191-MN586198	This study
NCFAD-2018-070-4	0704	2018-01-26	Windsor	Cluster 2 index case	MN586199-MN586206	This study
NCFAD-2018-070-5	0705	2018-02-01	Windsor	Cluster 2 index household	MN586207-MN586214	This study
NCFAD-2018-070-7	0707	2018-03-02	Orillia	Kerr kennel	MN586215-MN586222	This study
NCFAD-2018-070-8	0708	2018-03-02	Orillia	Kerr kennel	MN586223-MN586230	This study
NCFAD-2018-070-9	0709	2018-03-02	Orillia	Kerr kennel	MN586231-MN586238	This study
NCFAD-2018-070-10	07010	2018-03-09	Bracebridge	From neighbor’s dog that boarded at the rescue	MN586127-MN586134	This study
NCFAD-2018-070-11	07011	2018-03-09	Bracebridge	From neighbour’s dog	MN586135-MN586142	This study
NCFAD-2018-070-12	07012	2018-03-13	Colborne	Boarded	MN586143-MN586150	This study
NCFAD-2018-070-13	07013	2018-03-13	Colborne	Boarded	MN586151-MN586158	This study
NCFAD-2018-070-14	07014	2018-03-14	Colborne	Boarded	MN586159-MN586166	This study
NCFAD-2018-070-15	07015	2018-03-14	Colborne	Boarded	MN586167-MN586174	This study
NCFAD-2018-070-16	07016	2018-03-26	Colborne	Boarded	MN586175-MN586182	This study

### Phylogenetic analyses

The nucleotides in the coding regions of segments 1 (PB2), 4 (HA), 5 (NP), and 6 (NA) were aligned using Muscle in MEGA-X^23^. The full nucleotide sequences of segments 2 (PB1 and PB1-F2), 3 (PA, and PA-X), 7 (M1 and M2), and 8 (NS1 and NS2) were also aligned using Muscle, and the sequences were edited such that all of the codons in first open reading frame (ORF) were followed by the remaining codons in the second ORF in MEGA-X^23^. Rate of nucleotide substitution per site per year (subs/site/year) and the time to the most recent common ancestor (tMRCA) of 183 H3N2 CIV strains were estimated for each gene segment and a concatenation of the eight gene segments using the Bayesian Markov Chain Monte Carlo (BMCMC) method in the program BEAST, version2.5.2^15,16^. The best-fit nucleotide substitution model was determined by MEGA-X software^23^ and Hasegawa-Kishino-Yano (HKY)** +** G model was applied to each gene segment and the concatenated gene segment for Maximum Likelihood (ML) and Bayesian analyses. The age of the viruses was defined as the date of sample collection. The strict clock model and coalescent constant population for tree prior were used as they have been shown to best reflect the population dynamics of H3N2 CIV^6^. For the dataset, at least two independent BEAST analyses were run for 50 million generations, sampling every 5000 generations. Convergences and effective sample sizes (ESS) of the estimates were checked using Tracer v1.7.0 http://tree.bio.ed.ac.uk/software/tracer). All parameter estimates for each run showed ESS values > 200. A maximum clade credibility (MCC) phylogenetic tree was generated to summarize all 10,000 trees after a 10% burn-in using TreeAnnotator in BEAST^15,16^. The time-stamped phylogenetic tree was visualized and annotated using FigTreev1.4.4 (http://tree.bio.ed.ac.uk/software/figtree). The topologies of the MCC trees were compared to those inferred using the Maximum Likelihood (ML) method in the PhyML program^25^.

The concatenated eight gene segments of 21 Canadian CIV H3N2 strains were used to construct a phylogenetic network using the Median Joining method in the program Network v5.0.1.1 (http//www.fluxus-engineering.com). The parameter epsilon, which controls the level of homoplasy, was set at the same value as the weight of characters used to calculate the genetic distances (weight value = 10).

### Analysis of selection pressure

Site-specific selection pressures for all segments of the 183 H3N2 CIV strains were measured as nonsynonymous (dN) - synonymous (dS) nucleotide substitutions per site. In all cases, the differences were estimated using the SLAC (Single-Likelihood Ancestor Counting)^26^, FEL (Fixed Effects Likelihood)^26^, MEME (Mixed Effects Model of Evolution)^27^, and FUBAR (Fast, Unconstrained Bayesian AppRoximation)^28^ methods available at the Datamonkey^29^; http:// www.datamonkey.org/ online version of the HyPhy package^26^. A cut-off p-value to classify a site as positively or negatively selected was set at 0.1 for SLAC and 0.01 for FEL and MEME methods. The cut-off value for the posterior probability in the FUBAR method was set at 0.9 to reflect a positive or negative selection at a given site.

## Supplementary information

Figure S1, S2 and S3.

Supplementary Table S1.
