# Supplementary material for: Phylogenetic Inference of H3N2 Canine Influenza A Outbreak in Ontario, Canada in 2018
Source: Sci Rep. 2020 Apr 14;10:6309. doi: 10.1038/s41598-020-63278-z (PMC7156495; doi:10.1038/s41598-020-63278-z)
Supplement: Supplementary file 1 — Figure S1, S2 and S3. [file 41598_2020_63278_MOESM1_ESM.pdf]

Supplementary Materials for

Phylogenetic Inference of H3N2 Canine Influenza A Outbreak in Ontario, Canada in 2018

Wanhong Xu1, J Scott Weese2, Davor Ojkic3, Oliver Lung1, Katherine Handel1, Yohannes Berhane1, 4*

* Corresponding author. E-mail address: yohannes.berhane@canada.ca

This PDF file includes:

Figs. S1 to S3

Other Supplementary Material for this manuscript includes: Table S1 [spreadsheet (.xlsx)]

Fig. S1. Maximum Likelihood tree of the individual gene segment of Canadian H3N2 CIVs. Viruses are colored by their epidemiological clusters (yellow for cluster C1; brown for cluster C2; cyan for cluster C3a; green for cluster C3b; and blue for cluster C4). Branch lengths are scaled according to the numbers of nucleotide substitutions per site. The tree is midpoint rooted for clarity.

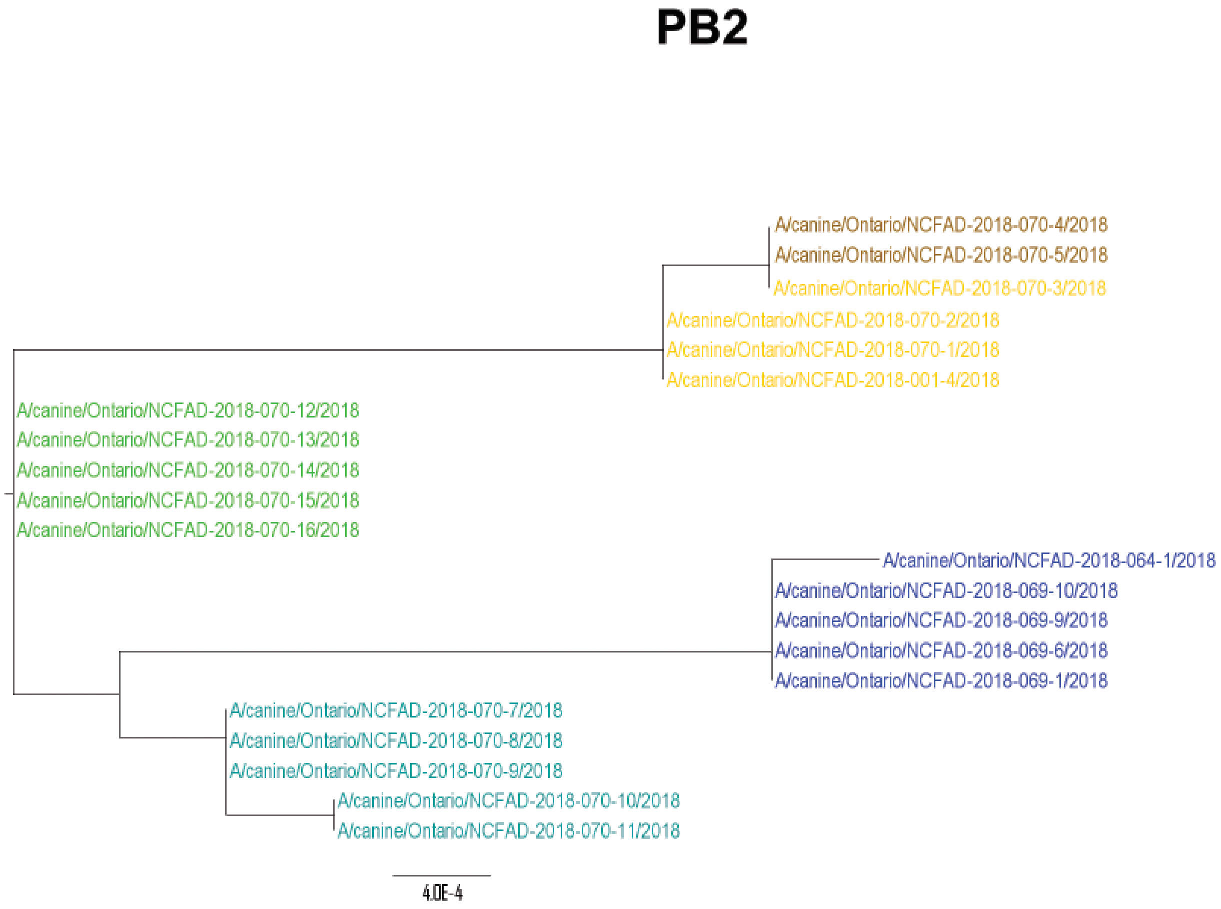

PB1

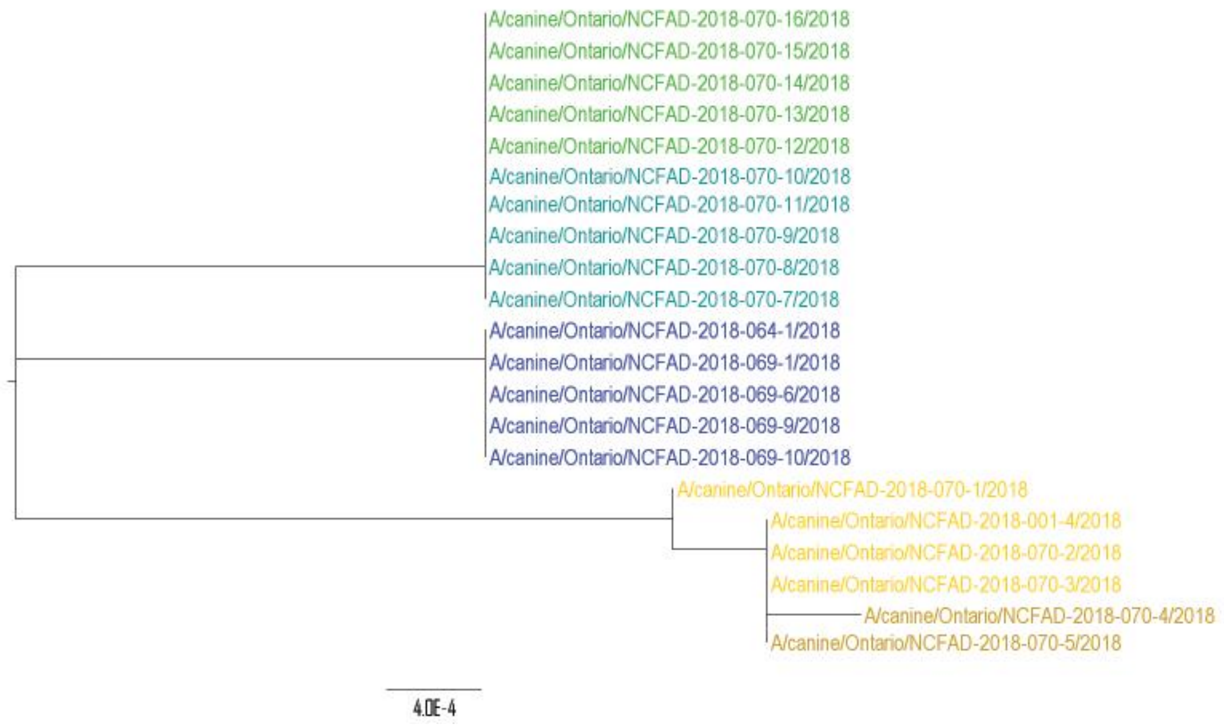
A/canine/Ontario/NCFAD-2018-070-16/2018
A/canine/Ontario/NCFAD-2018-070-15/2018
A/canine/Ontario/NCFAD-2018-070-14/2018
A/canine/Ontario/NCFAD-2018-070-13/2018
A/canine/Ontario/NCFAD-2018-070-12/2018
A/canine/Ontario/NCFAD-2018-070-10/2018
A/canine/Ontario/NCFAD-2018-070-11/2018
A/canine/Ontario/NCFAD-2018-070-9/2018
A/canine/Ontario/NCFAD-2018-070-8/2018
A/canine/Ontario/NCFAD-2018-070-7/2018
A/canine/Ontario/NCFAD-2018-064-1/2018
A/canine/Ontario/NCFAD-2018-069-1/2018
A/canine/Ontario/NCFAD-2018-069-6/2018
A/canine/Ontario/NCFAD-2018-069-9/2018
A/canine/Ontario/NCFAD-2018-069-10/2018
A/canine/Ontario/NCFAD-2018-070-1/2018
A/canine/Ontario/NCFAD-2018-001-4/2018
A/canine/Ontario/NCFAD-2018-070-2/2018
A/canine/Ontario/NCFAD-2018-070-3/2018
A/canine/Ontario/NCFAD-2018-070-4/2018
A/canine/Ontario/NCFAD-2018-070-5/2018
4.0E-4

PA

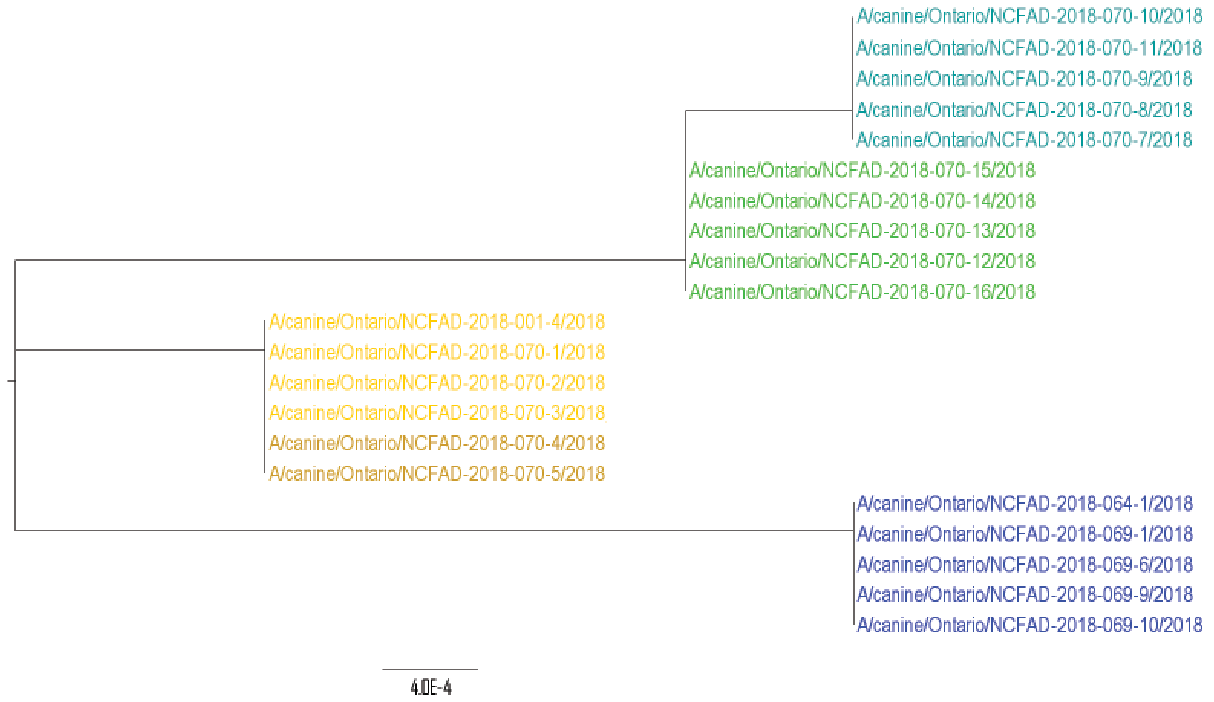
A/canine/Ontario/NCFAD-2018-070-10/2018
A/canine/Ontario/NCFAD-2018-070-11/2018
A/canine/Ontario/NCFAD-2018-070-9/2018
A/canine/Ontario/NCFAD-2018-070-8/2018
A/canine/Ontario/NCFAD-2018-070-7/2018
A/canine/Ontario/NCFAD-2018-070-15/2018
A/canine/Ontario/NCFAD-2018-070-14/2018
A/canine/Ontario/NCFAD-2018-070-13/2018
A/canine/Ontario/NCFAD-2018-070-12/2018
A/canine/Ontario/NCFAD-2018-070-16/2018
A/canine/Ontario/NCFAD-2018-001-4/2018
A/canine/Ontario/NCFAD-2018-070-1/2018
A/canine/Ontario/NCFAD-2018-070-2/2018
A/canine/Ontario/NCFAD-2018-070-3/2018
A/canine/Ontario/NCFAD-2018-070-4/2018
A/canine/Ontario/NCFAD-2018-070-5/2018
A/canine/Ontario/NCFAD-2018-064-1/2018
A/canine/Ontario/NCFAD-2018-069-1/2018
A/canine/Ontario/NCFAD-2018-069-6/2018
A/canine/Ontario/NCFAD-2018-069-9/2018
A/canine/Ontario/NCFAD-2018-069-10/2018
4.0E-4

HA

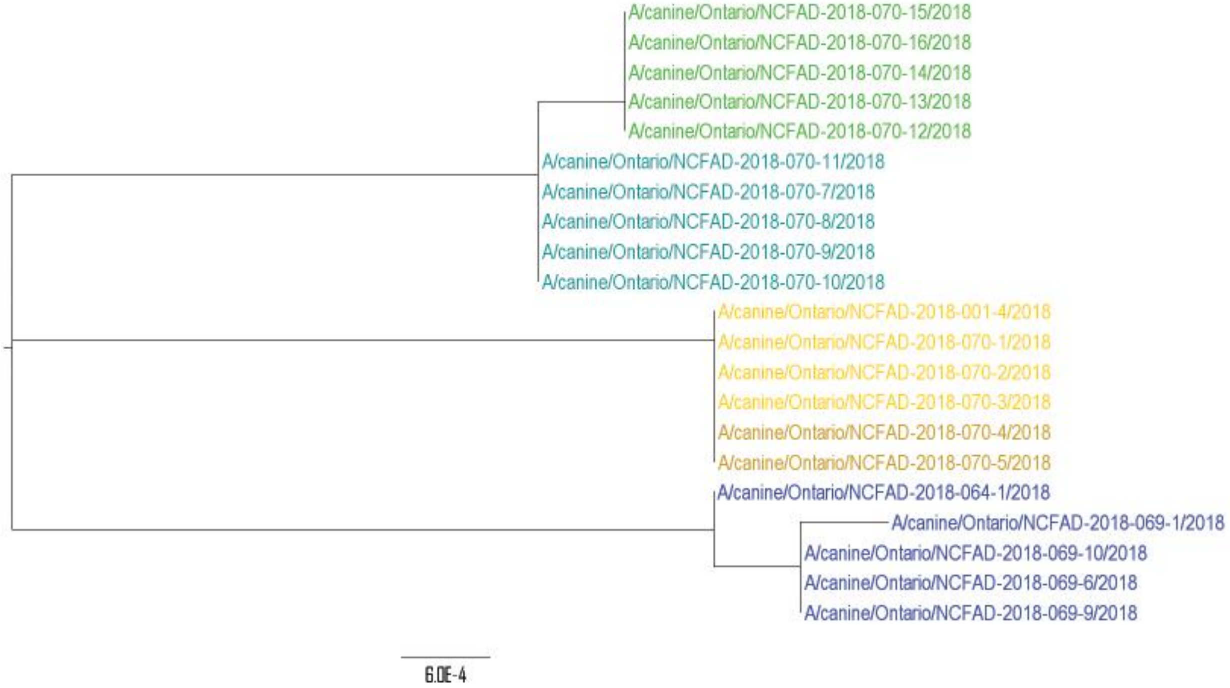
A/canine/Ontario/NCFAD-2018-070-15/2018
A/canine/Ontario/NCFAD-2018-070-16/2018
A/canine/Ontario/NCFAD-2018-070-14/2018
A/canine/Ontario/NCFAD-2018-070-13/2018
A/canine/Ontario/NCFAD-2018-070-12/2018
A/canine/Ontario/NCFAD-2018-070-11/2018
A/canine/Ontario/NCFAD-2018-070-7/2018
A/canine/Ontario/NCFAD-2018-070-8/2018
A/canine/Ontario/NCFAD-2018-070-9/2018
A/canine/Ontario/NCFAD-2018-070-10/2018
A/canine/Ontario/NCFAD-2018-001-4/2018
A/canine/Ontario/NCFAD-2018-070-1/2018
A/canine/Ontario/NCFAD-2018-070-2/2018
A/canine/Ontario/NCFAD-2018-070-3/2018
A/canine/Ontario/NCFAD-2018-070-4/2018
A/canine/Ontario/NCFAD-2018-070-5/2018
A/canine/Ontario/NCFAD-2018-064-1/2018
A/canine/Ontario/NCFAD-2018-069-1/2018
A/canine/Ontario/NCFAD-2018-069-10/2018
A/canine/Ontario/NCFAD-2018-069-6/2018
A/canine/Ontario/NCFAD-2018-069-9/2018
6.0E-4

NP

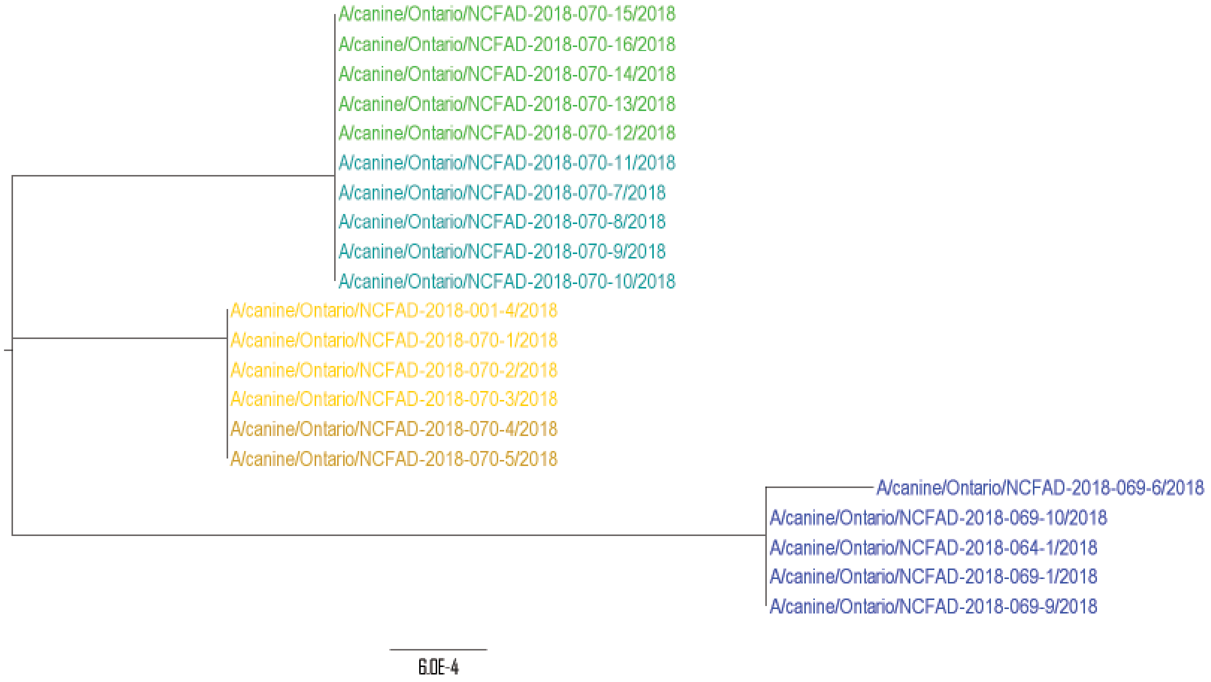

A/canine/Ontario/NCFAD-2018-070-15/2018
A/canine/Ontario/NCFAD-2018-070-16/2018
A/canine/Ontario/NCFAD-2018-070-14/2018
A/canine/Ontario/NCFAD-2018-070-13/2018
A/canine/Ontario/NCFAD-2018-070-12/2018
A/canine/Ontario/NCFAD-2018-070-11/2018
A/canine/Ontario/NCFAD-2018-070-7/2018
A/canine/Ontario/NCFAD-2018-070-8/2018
A/canine/Ontario/NCFAD-2018-070-9/2018
A/canine/Ontario/NCFAD-2018-070-10/2018
A/canine/Ontario/NCFAD-2018-001-4/2018
A/canine/Ontario/NCFAD-2018-070-1/2018
A/canine/Ontario/NCFAD-2018-070-2/2018
A/canine/Ontario/NCFAD-2018-070-3/2018
A/canine/Ontario/NCFAD-2018-070-4/2018
A/canine/Ontario/NCFAD-2018-070-5/2018
A/canine/Ontario/NCFAD-2018-069-6/2018
A/canine/Ontario/NCFAD-2018-069-10/2018
A/canine/Ontario/NCFAD-2018-064-1/2018
A/canine/Ontario/NCFAD-2018-069-1/2018
A/canine/Ontario/NCFAD-2018-069-9/2018
6.0E-4

NA

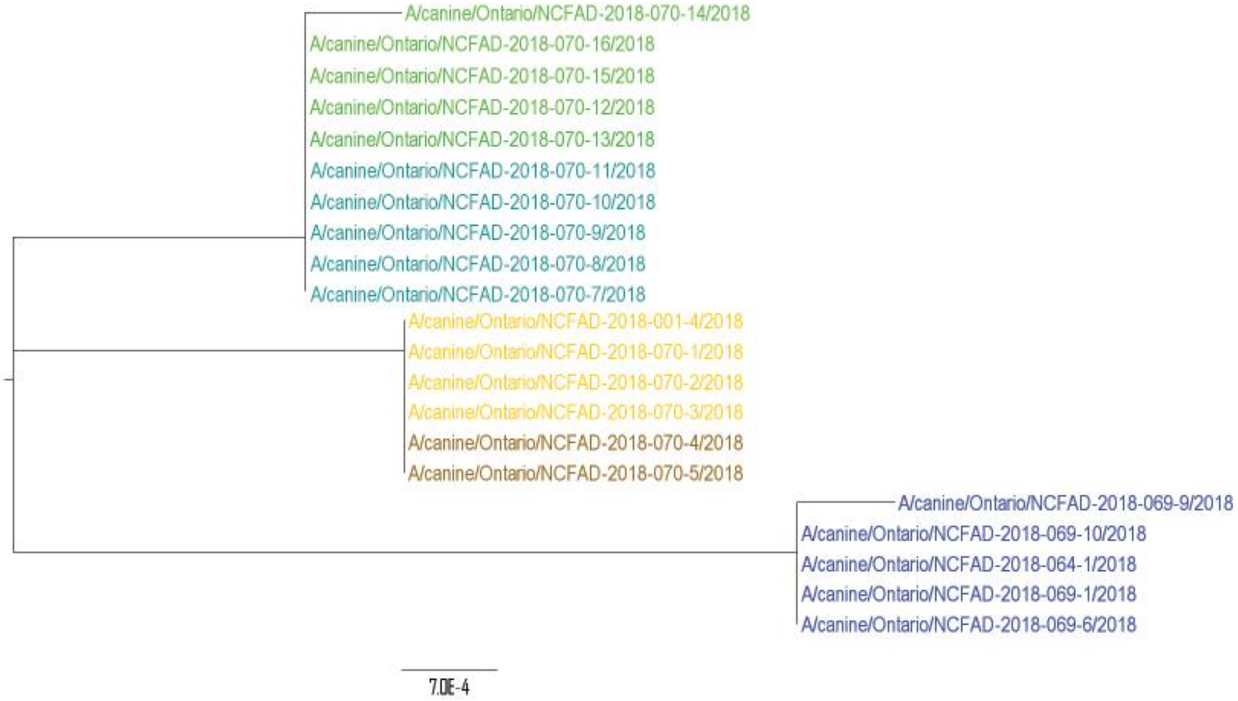
A/canine/Ontario/NCFAD-2018-070-14/2018
A/canine/Ontario/NCFAD-2018-070-16/2018
A/canine/Ontario/NCFAD-2018-070-15/2018
A/canine/Ontario/NCFAD-2018-070-12/2018
A/canine/Ontario/NCFAD-2018-070-13/2018
A/canine/Ontario/NCFAD-2018-070-11/2018
A/canine/Ontario/NCFAD-2018-070-10/2018
A/canine/Ontario/NCFAD-2018-070-9/2018
A/canine/Ontario/NCFAD-2018-070-8/2018
A/canine/Ontario/NCFAD-2018-070-7/2018
A/canine/Ontario/NCFAD-2018-001-4/2018
A/canine/Ontario/NCFAD-2018-070-1/2018
A/canine/Ontario/NCFAD-2018-070-2/2018
A/canine/Ontario/NCFAD-2018-070-3/2018
A/canine/Ontario/NCFAD-2018-070-4/2018
A/canine/Ontario/NCFAD-2018-070-5/2018
A/canine/Ontario/NCFAD-2018-069-9/2018
A/canine/Ontario/NCFAD-2018-069-10/2018
A/canine/Ontario/NCFAD-2018-064-1/2018
A/canine/Ontario/NCFAD-2018-069-1/2018
A/canine/Ontario/NCFAD-2018-069-6/2018
7.0E-4

M

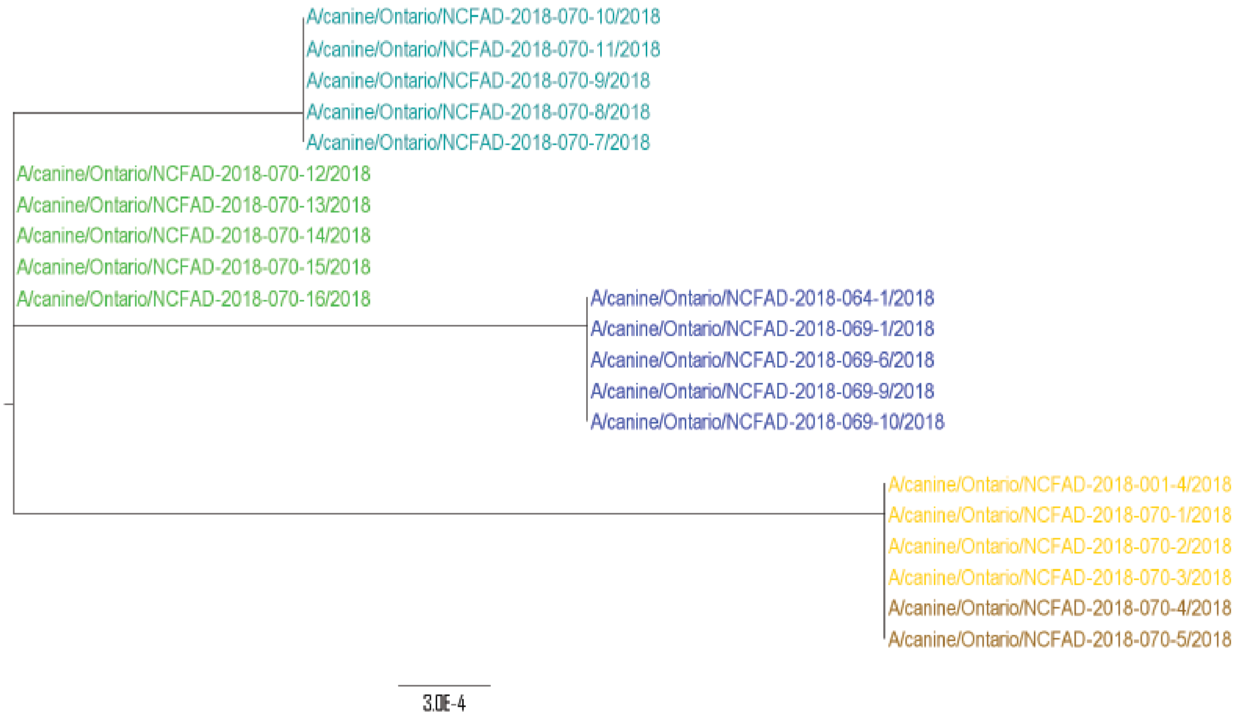
A/canine/Ontario/NCFAD-2018-070-10/2018
A/canine/Ontario/NCFAD-2018-070-11/2018
A/canine/Ontario/NCFAD-2018-070-9/2018
A/canine/Ontario/NCFAD-2018-070-8/2018
A/canine/Ontario/NCFAD-2018-070-7/2018
A/canine/Ontario/NCFAD-2018-070-12/2018
A/canine/Ontario/NCFAD-2018-070-13/2018
A/canine/Ontario/NCFAD-2018-070-14/2018
A/canine/Ontario/NCFAD-2018-070-15/2018
A/canine/Ontario/NCFAD-2018-070-16/2018
A/canine/Ontario/NCFAD-2018-064-1/2018
A/canine/Ontario/NCFAD-2018-069-1/2018
A/canine/Ontario/NCFAD-2018-069-6/2018
A/canine/Ontario/NCFAD-2018-069-9/2018
A/canine/Ontario/NCFAD-2018-069-10/2018
A/canine/Ontario/NCFAD-2018-001-4/2018
A/canine/Ontario/NCFAD-2018-070-1/2018
A/canine/Ontario/NCFAD-2018-070-2/2018
A/canine/Ontario/NCFAD-2018-070-3/2018
A/canine/Ontario/NCFAD-2018-070-4/2018
A/canine/Ontario/NCFAD-2018-070-5/2018
3.0E-4

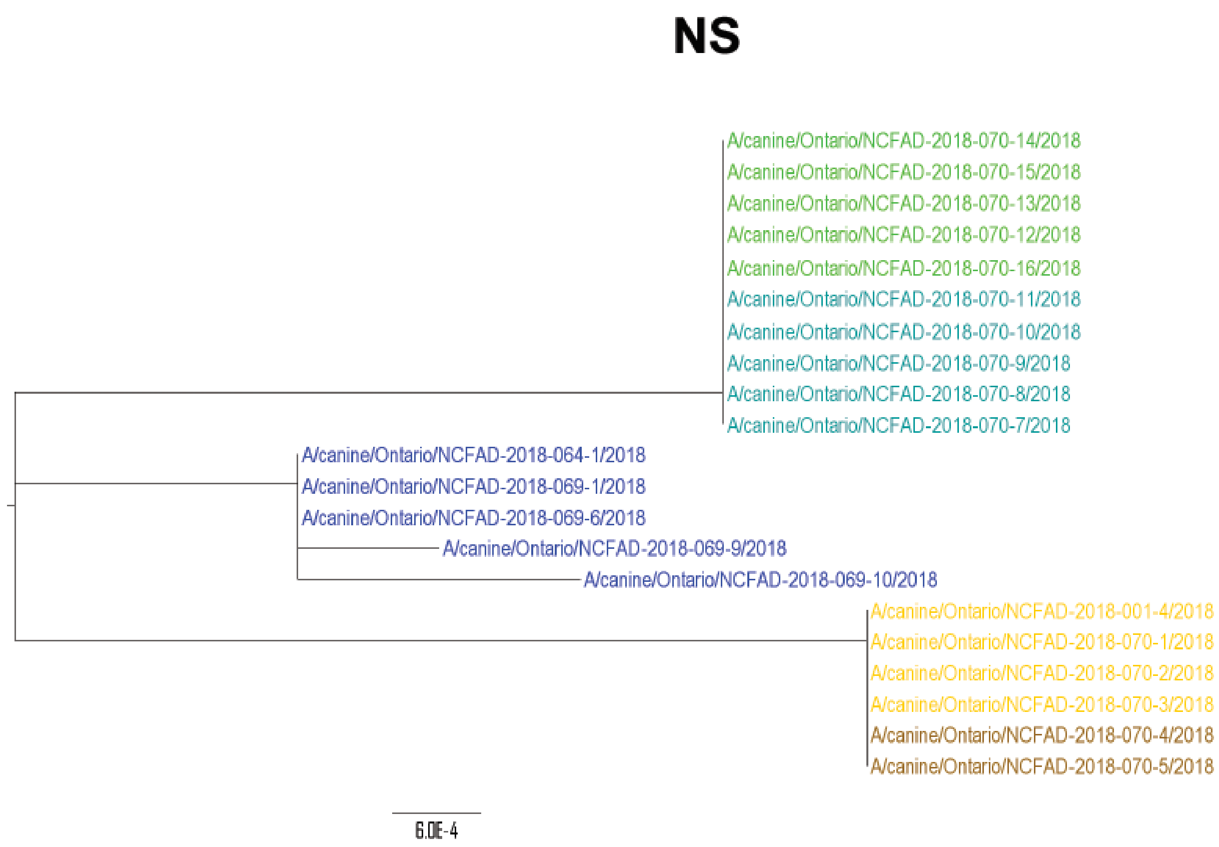
NS
A/canine/Ontario/NCFAD-2018-070-14/2018
A/canine/Ontario/NCFAD-2018-070-15/2018
A/canine/Ontario/NCFAD-2018-070-13/2018
A/canine/Ontario/NCFAD-2018-070-12/2018
A/canine/Ontario/NCFAD-2018-070-16/2018
A/canine/Ontario/NCFAD-2018-070-11/2018
A/canine/Ontario/NCFAD-2018-070-10/2018
A/canine/Ontario/NCFAD-2018-070-9/2018
A/canine/Ontario/NCFAD-2018-070-8/2018
A/canine/Ontario/NCFAD-2018-070-7/2018
A/canine/Ontario/NCFAD-2018-064-1/2018
A/canine/Ontario/NCFAD-2018-069-1/2018
A/canine/Ontario/NCFAD-2018-069-6/2018
A/canine/Ontario/NCFAD-2018-069-9/2018
A/canine/Ontario/NCFAD-2018-069-10/2018
A/canine/Ontario/NCFAD-2018-001-4/2018
A/canine/Ontario/NCFAD-2018-070-1/2018
A/canine/Ontario/NCFAD-2018-070-2/2018
A/canine/Ontario/NCFAD-2018-070-3/2018
A/canine/Ontario/NCFAD-2018-070-4/2018
A/canine/Ontario/NCFAD-2018-070-5/2018
6.0E-4

Fig. S2. Maximum clade credibility tree. Time-scaled phylogenies (dates on the horizontal axis) inferred using Bayesian MCMC analysis. Denotations are the same as in Fig. S1. Phylogenetic clusters are denoted as I to IV.

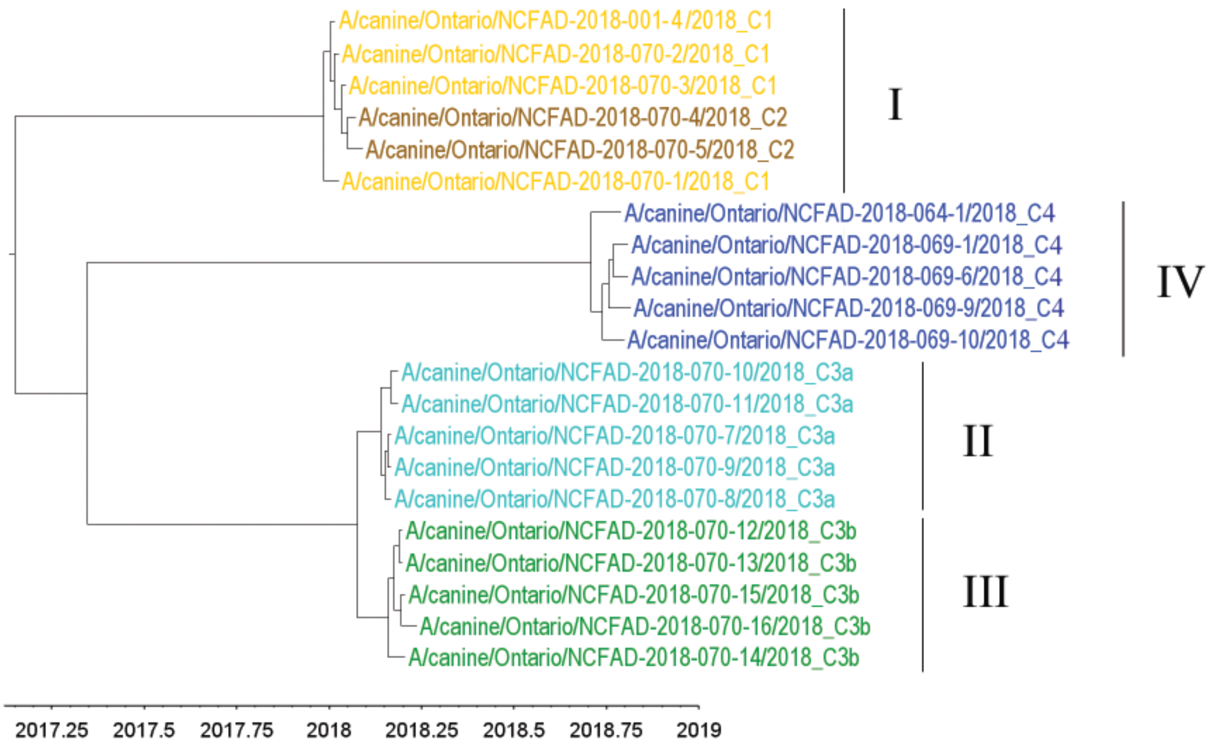

Fig. S3. Maximum clade credibility tree inferred for the individual gene segment of 183 H3N2 CIVs. Sequences are colored according to the country of origin. Red, Canada; blue, the United States; green, China; brown, South Korea.

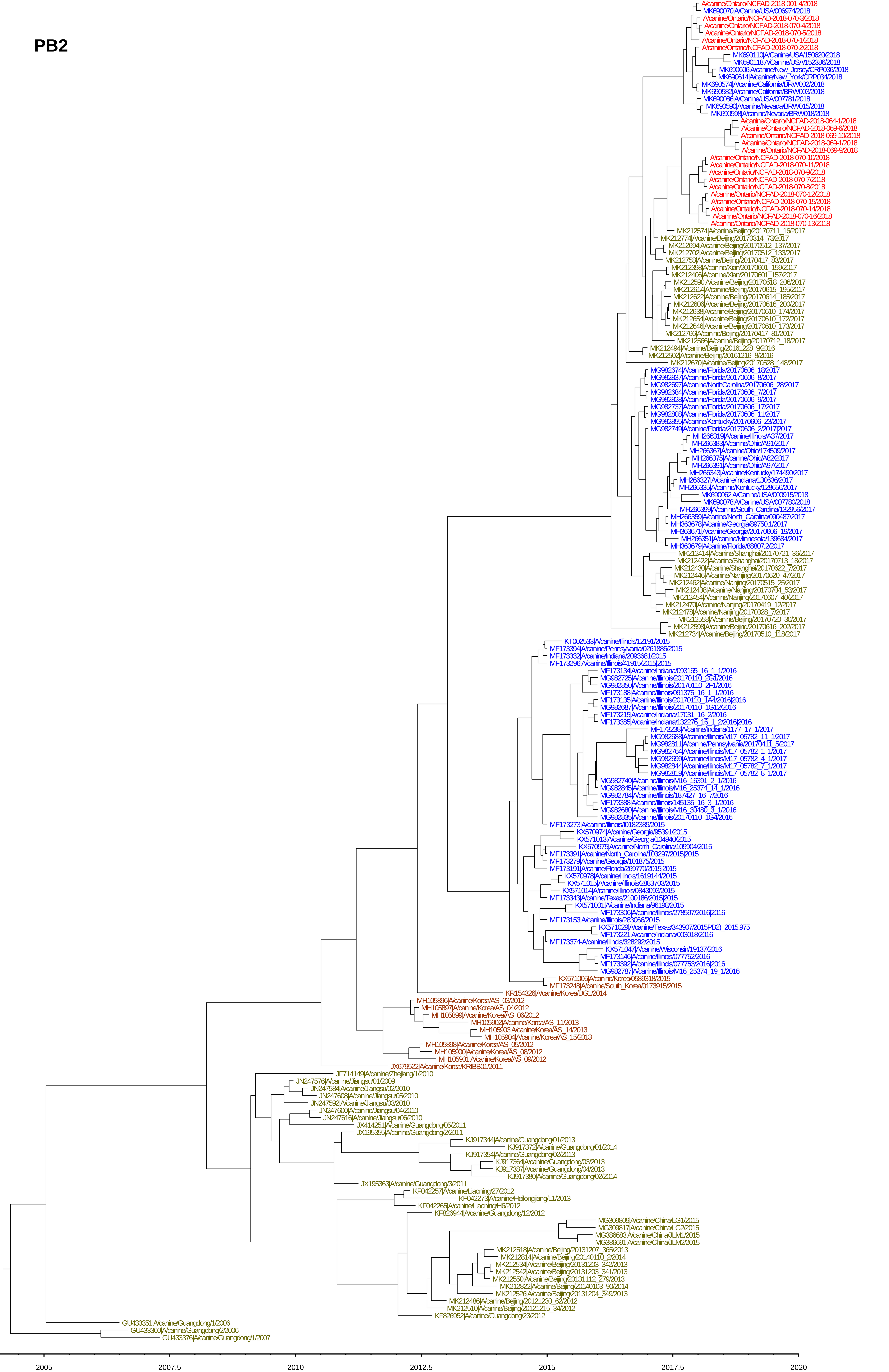
PB2
A/canine/Ontario/NCFAD-2018-001-4/2018
MK690070|A/Canine/USA/006974/2018
A/canine/Ontario/NCFAD-2018-070-3/2018
A/canine/Ontario/NCFAD-2018-070-4/2018
A/canine/Ontario/NCFAD-2018-070-5/2018
A/canine/Ontario/NCFAD-2018-070-1/2018
A/canine/Ontario/NCFAD-2018-070-2/2018
MK690110|A/Canine/USA/150620/2018
MK690118|A/Canine/USA/152386/2018
MK690606|A/canine/New_Jersey/CRP036/2018
MK690614|A/canine/New_York/CRP034/2018
MK690574|A/canine/California/BRW002/2018
MK690582|A/canine/California/BRW003/2018
MK690086|A/Canine/USA/007781/2018
MK690590|A/canine/Nevada/BRW015/2018
MK690598|A/canine/Nevada/BRW018/2018
A/canine/Ontario/NCFAD-2018-064-1/2018
A/canine/Ontario/NCFAD-2018-069-6/2018
A/canine/Ontario/NCFAD-2018-069-10/2018
A/canine/Ontario/NCFAD-2018-069-1/2018
A/canine/Ontario/NCFAD-2018-069-9/2018
A/canine/Ontario/NCFAD-2018-070-10/2018
A/canine/Ontario/NCFAD-2018-070-11/2018
A/canine/Ontario/NCFAD-2018-070-9/2018
A/canine/Ontario/NCFAD-2018-070-7/2018
A/canine/Ontario/NCFAD-2018-070-8/2018
A/canine/Ontario/NCFAD-2018-070-12/2018
A/canine/Ontario/NCFAD-2018-070-15/2018
A/canine/Ontario/NCFAD-2018-070-14/2018
A/canine/Ontario/NCFAD-2018-070-16/2018
A/canine/Ontario/NCFAD-2018-070-13/2018
MK212574|A/canine/Beijing/20170711_16/2017
MK212774|A/canine/Beijing/20170314_73/2017
MK212694|A/canine/Beijing/20170512_137/2017
MK212702|A/canine/Beijing/20170512_133/2017
MK212758|A/canine/Beijing/20170417_83/2017
MK212398|A/canine/Xian/20170601_159/2017
MK212406|A/canine/Xian/20170601_157/2017
MK212590|A/canine/Beijing/20170618_206/2017
MK212614|A/canine/Beijing/20170615_195/2017
MK212622|A/canine/Beijing/20170614_185/2017
MK212606|A/canine/Beijing/20170616_200/2017
MK212638|A/canine/Beijing/20170610_174/2017
MK212654|A/canine/Beijing/20170610_172/2017
MK212646|A/canine/Beijing/20170610_173/2017
MK212766|A/canine/Beijing/20170417_81/2017
MK212566|A/canine/Beijing/20170712_18/2017
MK212494|A/canine/Beijing/20161228_9/2016
MK212502|A/canine/Beijing/20161216_8/2016
MK212670|A/canine/Beijing/20170528_148/2017
MG982674|A/canine/Florida/20170606_18/2017
MG982837|A/canine/Florida/20170606_8/2017
MG982697|A/canine/NorthCarolina/20170606_28/2017
MG982684|A/canine/Florida/20170606_7/2017
MG982828|A/canine/Florida/20170606_9/2017
MG982737|A/canine/Florida/20170606_17/2017
MG982808|A/canine/Florida/20170606_11/2017
MG982855|A/canine/Kentucky/20170606_23/2017
MG982749|A/canine/Florida/20170606_2/2017|2017
MH266319|A/canine/Illinois/A37/2017
MH266383|A/canine/Ohio/A91/2017
MH266367|A/canine/Ohio/174509/2017
MH266375|A/canine/Ohio/A82/2017
MH266391|A/canine/Ohio/A97/2017
MH266343|A/canine/Kentucky/174490/2017
MH266327|A/canine/Indiana/130636/2017
MH266335|A/canine/Kentucky/128656/2017
MK690062|A/Canine/USA/000915/2018
MK690078|A/Canine/USA/007780/2018
MH266399|A/canine/South_Carolina/132956/2017
MH266359|A/canine/North_Carolina/090487/2017
MH363678|A/canine/Georgia/89750.1/2017
MH363671|A/canine/Georgia/20170606_19/2017
MH266351|A/canine/Minnesota/139684/2017
MH363679|A/canine/Florida/88807.2/2017
MK212414|A/canine/Shanghai/20170721_36/2017
MK212422|A/canine/Shanghai/20170713_18/2017
MK212430|A/canine/Shanghai/20170622_7/2017
MK212446|A/canine/Nanjing/20170620_47/2017
MK212462|A/canine/Nanjing/20170515_25/2017
MK212438|A/canine/Nanjing/20170704_53/2017
MK212454|A/canine/Nanjing/20170607_40/2017
MK212470|A/canine/Nanjing/20170419_12/2017
MK212478|A/canine/Nanjing/20170328_7/2017
MK212558|A/canine/Beijing/20170720_30/2017
MK212598|A/canine/Beijing/20170616_202/2017
MK212734|A/canine/Beijing/20170510_118/2017
KT002533|A/canine/Illinois/12191/2015
MF173394|A/canine/Pennsylvania/0261885/2015
MF173332|A/canine/Indiana/2093681/2015
MF173296|A/canine/Illinois/41915/2015|2015
MF173134|A/canine/Indiana/093165_16_1_1/2016
MG982725|A/canine/Illinois/20170110_2G1/2016
MG982850|A/canine/Illinois/20170110_2F1/2016
MF173188|A/canine/Illinois/091375_16_1_1/2016
MF173135|A/canine/Illinois/20170110_1A4/2016|2016
MG982687|A/canine/Illinois/20170110_1G12/2016
MF173215|A/canine/Indiana/17031_16_2/2016
MF173385|A/canine/Indiana/132276_16_1_2/2016|2016
MF173238|A/canine/Indiana/1177_17_1/2017
MG982688|A/canine/Illinois/M17_05782_11_1/2017
MG982811|A/canine/Pennsylvania/20170411_5/2017
MG982764|A/canine/Illinois/M17_05782_1_1/2017
MG982699|A/canine/Illinois/M17_05782_4_1/2017
MG982844|A/canine/Illinois/M17_05782_7_1/2017
MG982819|A/canine/Illinois/M17_05782_8_1/2017
MG982740|A/canine/Illinois/M16_16391_2_1/2016
MG982845|A/canine/Illinois/M16_25374_14_1/2016
MG982784|A/canine/Illinois/187427_16_7/2016
MF173388|A/canine/Illinois/145135_16_3_1/2016
MG982680|A/canine/Illinois/M16_30480_3_1/2016
MG982835|A/canine/Illinois/20170110_1G4/2016
MF173273|A/canine/Illinois/I0182389/2015
KX570974|A/canine/Georgia/95391/2015
KX571013|A/canine/Georgia/104940/2015
KX570975|A/canine/North_Carolina/109904/2015
MF173391|A/canine/North_Carolina/103297/2015|2015
MF173279|A/canine/Georgia/101875/2015
MF173191|A/canine/Florida/269770/2015|2015
KX570978|A/canine/Illinois/1619144/2015
KX571015|A/canine/Illinois/2883703/2015
KX571014|A/canine/Illinois/0843093/2015
MF173343|A/canine/Texas/2100186/2015|2015
KX571001|A/canine/Indiana/96198/2015
MF173306|A/canine/Illinois/278597/2016|2016
MF173153|A/canine/Illinois/283066/2015
KX571029|A/canine/Texas/343907/2015PB2)_2015.975
MF173221|A/canine/Indiana/003018/2016
MF173374-A/canine/Illinois/328292/2015
KX571047|A/canine/Wisconsin/19137/2016
MF173146|A/canine/Illinois/077752/2016
MF173392|A/canine/Illinois/077753/2016|2016
MG982787|A/canine/Illinois/M16_25374_19_1/2016
KX571005|A/canine/Korea/0589318/2015
MF173248|A/canine/South_Korea/0173915/2015
KR154326|A/canine/Korea/DG1/2014
MH105896|A/canine/Korea/AS_03/2012
MH105897|A/canine/Korea/AS_04/2012
MH105899|A/canine/Korea/AS_06/2012
MH105902|A/canine/Korea/AS_11/2013
MH105903|A/canine/Korea/AS_14/2013
MH105904|A/canine/Korea/AS_15/2013
MH105898|A/canine/Korea/AS_05/2012
MH105900|A/canine/Korea/AS_08/2012
MH105901|A/canine/Korea/AS_09/2012
JX679522|A/canine/Korea/KRIBB01/2011
JF714149|A/canine/Zhejiang/1/2010
JN247576|A/canine/Jiangsu/01/2009
JN247584|A/canine/Jiangsu/02/2010
JN247608|A/canine/Jiangsu/05/2010
JN247592|A/canine/Jiangsu/03/2010
JN247600|A/canine/Jiangsu/04/2010
JN247616|A/canine/Jiangsu/06/2010
JX414251|A/canine/Guangdong/05/2011
JX195355|A/canine/Guangdong/2/2011
KJ917344|A/canine/Guangdong/01/2013
KJ917372|A/canine/Guangdong/01/2014
KJ917354|A/canine/Guangdong/02/2013
KJ917364|A/canine/Guangdong/03/2013
KJ917367|A/canine/Guangdong/04/2013
KJ917380|A/canine/Guangdong/02/2014
JX195363|A/canine/Guangdong/3/2011
KF042257|A/canine/Liaoning/27/2012
KF042273|A/canine/Heilongjiang/L1/2013
KF042265|A/canine/Liaoning/H6/2012
KF826944|A/canine/Guangdong/12/2012
MG309809|A/canine/China/LG1/2015
MG309817|A/canine/China/LG2/2015
MG386683|A/canine/China/JLM1/2015
MG386691|A/canine/China/JLM2/2015
MK212518|A/canine/Beijing/20131207_365/2013
MK212814|A/canine/Beijing/20140110_2/2014
MK212534|A/canine/Beijing/20131203_342/2013
MK212542|A/canine/Beijing/20131203_341/2013
MK212550|A/canine/Beijing/20131112_279/2013
MK212822|A/canine/Beijing/20140103_90/2014
MK212526|A/canine/Beijing/20131204_349/2013
MK212486|A/canine/Beijing/20121230_62/2012
MK212510|A/canine/Beijing/20121215_34/2012
KF826952|A/canine/Guangdong/23/2012
GU433351|A/canine/Guangdong/1/2006
GU433360|A/canine/Guangdong/2/2006
GU433376|A/canine/Guangdong/1/2007
2005
2007.5
2010
2012.5
2015
2017.5
2020

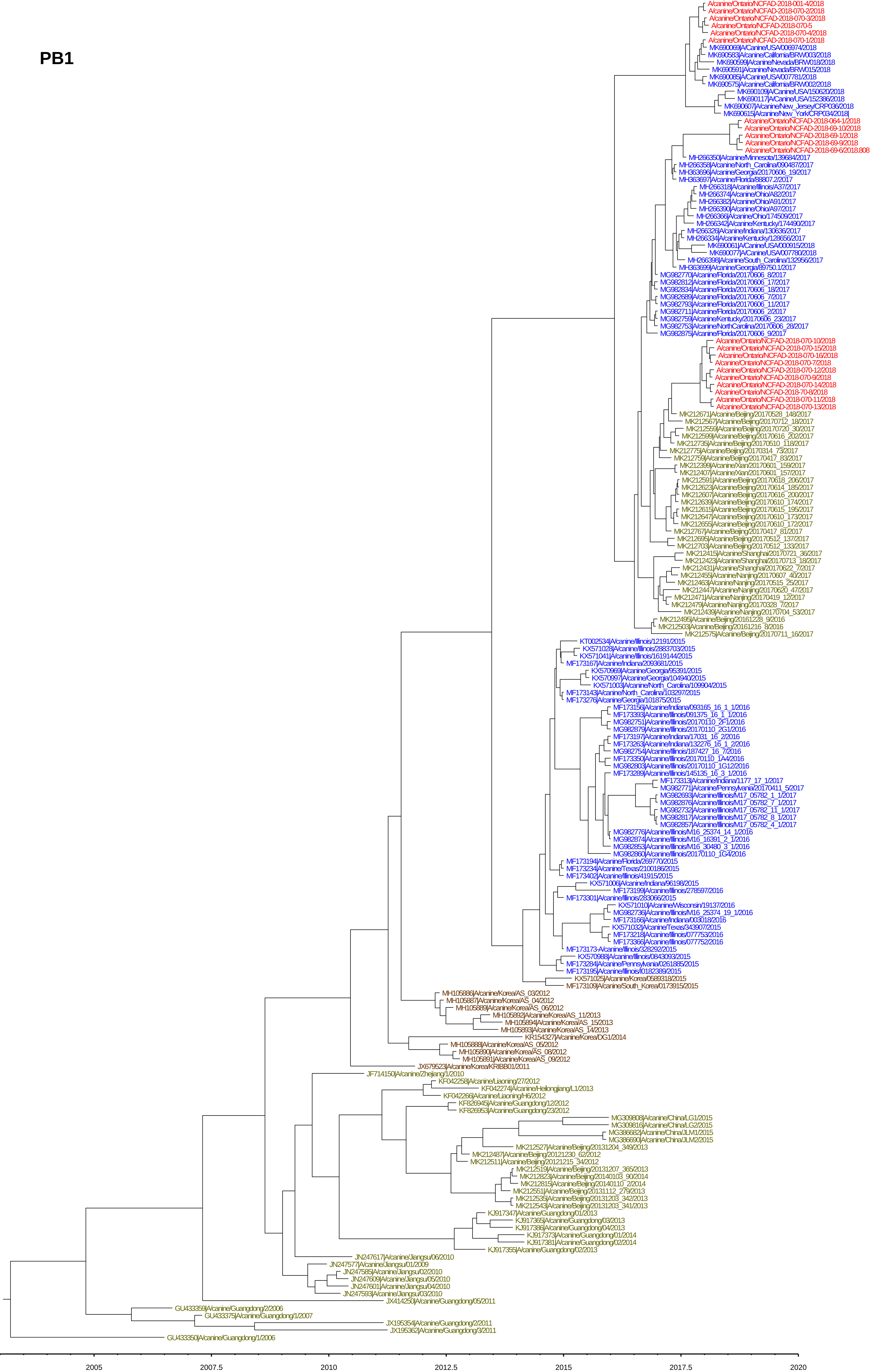

PB1
A/canine/Ontario/NCFAD-2018-001-4/2018
A/canine/Ontario/NCFAD-2018-070-2/2018
A/canine/Ontario/NCFAD-2018-070-3/2018
A/canine/Ontario/NCFAD-2018-070-5
A/canine/Ontario/NCFAD-2018-070-4/2018
A/canine/Ontario/NCFAD-2018-070-1/2018
MK690069|A/Canine/USA/006974/2018
MK690583|A/canine/California/BRW003/2018
MK690599|A/canine/Nevada/BRW018/2018
MK690591|A/canine/Nevada/BRW015/2018
MK690085|A/Canine/USA/007781/2018
MK690575|A/canine/California/BRW002/2018
MK690109|A/Canine/USA/150620/2018
MK690117|A/Canine/USA/152386/2018
MK690607|A/canine/New_Jersey/CRP036/2018
MK690615|A/canine/New_York/CRP034/2018|
A/canine/Ontario/NCFAD-2018-064-1/2018
A/canine/Ontario/NCFAD-2018-69-10/2018
A/canine/Ontario/NCFAD-2018-69-1/2018
A/canine/Ontario/NCFAD-2018-69-9/2018
A/canine/Ontario/NCFAD-2018-69-6/2018.808
MH266350|A/canine/Minnesota/139684/2017
MH266358|A/canine/North_Carolina/090487/2017
MH363696|A/canine/Georgia/20170606_19/2017
MH363697|A/canine/Florida/88807.2/2017
MH266318|A/canine/Illinois/A37/2017
MH266374|A/canine/Ohio/A82/2017
MH266382|A/canine/Ohio/A91/2017
MH266390|A/canine/Ohio/A97/2017
MH266366|A/canine/Ohio/174509/2017
MH266342|A/canine/Kentucky/174490/2017
MH266326|A/canine/Indiana/130636/2017
MH266334|A/canine/Kentucky/128656/2017
MK690061|A/Canine/USA/000915/2018
MK690077|A/Canine/USA/007780/2018
MH266398|A/canine/South_Carolina/132956/2017
MH363699|A/canine/Georgia/89750.1/2017
MG982770|A/canine/Florida/20170606_8/2017
MG982812|A/canine/Florida/20170606_17/2017
MG982834|A/canine/Florida/20170606_18/2017
MG982689|A/canine/Florida/20170606_7/2017
MG982793|A/canine/Florida/20170606_11/2017
MG982711|A/canine/Florida/20170606_2/2017
MG982759|A/canine/Kentucky/20170606_23/2017
MG982753|A/canine/NorthCarolina/20170606_28/2017
MG982875|A/canine/Florida/20170606_9/2017
A/canine/Ontario/NCFAD-2018-070-10/2018
A/canine/Ontario/NCFAD-2018-070-15/2018
A/canine/Ontario/NCFAD-2018-070-16/2018
A/canine/Ontario/NCFAD-2018-070-7/2018
A/canine/Ontario/NCFAD-2018-070-12/2018
A/canine/Ontario/NCFAD-2018-070-9/2018
A/canine/Ontario/NCFAD-2018-070-14/2018
A/canine/Ontario/NCFAD-2018-70-8/2018
A/canine/Ontario/NCFAD-2018-070-11/2018
A/canine/Ontario/NCFAD-2018-070-13/2018
MK212671|A/canine/Beijing/20170528_148/2017
MK212567|A/canine/Beijing/20170712_18/2017
MK212559|A/canine/Beijing/20170720_30/2017
MK212599|A/canine/Beijing/20170616_202/2017
MK212735|A/canine/Beijing/20170510_118/2017
MK212775|A/canine/Beijing/20170314_73/2017
MK212759|A/canine/Beijing/20170417_83/2017
MK212399|A/canine/Xian/20170601_159/2017
MK212407|A/canine/Xian/20170601_157/2017
MK212591|A/canine/Beijing/20170618_206/2017
MK212623|A/canine/Beijing/20170614_185/2017
MK212607|A/canine/Beijing/20170616_200/2017
MK212639|A/canine/Beijing/20170610_174/2017
MK212615|A/canine/Beijing/20170615_195/2017
MK212647|A/canine/Beijing/20170610_173/2017
MK212655|A/canine/Beijing/20170610_172/2017
MK212767|A/canine/Beijing/20170417_81/2017
MK212695|A/canine/Beijing/20170512_137/2017
MK212703|A/canine/Beijing/20170512_133/2017
MK212415|A/canine/Shanghai/20170721_36/2017
MK212423|A/canine/Shanghai/20170713_18/2017
MK212431|A/canine/Shanghai/20170622_7/2017
MK212455|A/canine/Nanjing/20170607_40/2017
MK212463|A/canine/Nanjing/20170515_25/2017
MK212447|A/canine/Nanjing/20170620_47/2017
MK212471|A/canine/Nanjing/20170419_12/2017
MK212479|A/canine/Nanjing/20170328_7/2017
MK212439|A/canine/Nanjing/20170704_53/2017
MK212495|A/canine/Beijing/20161228_9/2016
MK212503|A/canine/Beijing/20161216_8/2016
MK212575|A/canine/Beijing/20170711_16/2017
KT002534|A/canine/Illinois/12191/2015
KX571028|A/canine/Illinois/2883703/2015
KX571041|A/canine/Illinois/1619144/2015
MF173167|A/canine/Indiana/2093681/2015
KX570969|A/canine/Georgia/95391/2015
KX570997|A/canine/Georgia/104940/2015
KX571003|A/canine/North_Carolina/109904/2015
MF173143|A/canine/North_Carolina/103297/2015
MF173276|A/canine/Georgia/101875/2015
MF173156|A/canine/Indiana/093165_16_1_1/2016
MF173393|A/canine/Illinois/091375_16_1_1/2016
MG982751|A/canine/Illinois/20170110_2F1/2016
MG982879|A/canine/Illinois/20170110_2G1/2016
MF173197|A/canine/Indiana/17031_16_2/2016
MF173263|A/canine/Indiana/132276_16_1_2/2016
MG982754|A/canine/Illinois/187427_16_7/2016
MF173350|A/canine/Illinois/20170110_1A4/2016
MG982803|A/canine/Illinois/20170110_1G12/2016
MF173289|A/canine/Illinois/145135_16_3_1/2016
MF173313|A/canine/Indiana/1177_17_1/2017
MG982771|A/canine/Pennsylvania/20170411_5/2017
MG982693|A/canine/Illinois/M17_05782_1_1/2017
MG982876|A/canine/Illinois/M17_05782_7_1/2017
MG982732|A/canine/Illinois/M17_05782_11_1/2017
MG982817|A/canine/Illinois/M17_05782_8_1/2017
MG982857|A/canine/Illinois/M17_05782_4_1/2017
MG982776|A/canine/Illinois/M16_25374_14_1/2016
MG982874|A/canine/Illinois/M16_16391_2_1/2016
MG982853|A/canine/Illinois/M16_30480_3_1/2016
MG982860|A/canine/Illinois/20170110_1G4/2016
MF173194|A/canine/Florida/269770/2015
MF173234|A/canine/Texas/2100186/2015
MF173402|A/canine/Illinois/41915/2015
KX571006|A/canine/Indiana/96198/2015
MF173199|A/canine/Illinois/278597/2016
MF173301|A/canine/Illinois/283066/2015
KX571010|A/canine/Wisconsin/19137/2016
MG982736|A/canine/Illinois/M16_25374_19_1/2016
MF173166|A/canine/Indiana/003018/2016
KX571032|A/canine/Texas/343907/2015
MF173218|A/canine/Illinois/077753/2016
MF173366|A/canine/Illinois/077752/2016
MF173173-A/canine/Illinois/328292/2015
KX570988|A/canine/Illinois/0843093/2015
MF173284|A/canine/Pennsylvania/0261885/2015
MF173195|A/canine/Illinois/I0182389/2015
KX571025|A/canine/Korea/0589318/2015
MF173109|A/canine/South_Korea/0173915/2015
MH105886|A/canine/Korea/AS_03/2012
MH105887|A/canine/Korea/AS_04/2012
MH105889|A/canine/Korea/AS_06/2012
MH105892|A/canine/Korea/AS_11/2013
MH105894|A/canine/Korea/AS_15/2013
MH105893|A/canine/Korea/AS_14/2013
KR154327|A/canine/Korea/DG1/2014
MH105888|A/canine/Korea/AS_05/2012
MH105890|A/canine/Korea/AS_08/2012
MH105891|A/canine/Korea/AS_09/2012
JX679523|A/canine/Korea/KRIBB01/2011
JF714150|A/canine/Zhejiang/1/2010
KF042258|A/canine/Liaoning/27/2012
KF042274|A/canine/Heilongjiang/L1/2013
KF042266|A/canine/Liaoning/H6/2012
KF826945|A/canine/Guangdong/12/2012
KF826953|A/canine/Guangdong/23/2012
MG309808|A/canine/China/LG1/2015
MG309816|A/canine/China/LG2/2015
MG386682|A/canine/China/JLM1/2015
MG386690|A/canine/China/JLM2/2015
MK212527|A/canine/Beijing/20131204_349/2013
MK212487|A/canine/Beijing/20121230_62/2012
MK212511|A/canine/Beijing/20121215_34/2012
MK212519|A/canine/Beijing/20131207_365/2013
MK212823|A/canine/Beijing/20140103_90/2014
MK212815|A/canine/Beijing/20140110_2/2014
MK212551|A/canine/Beijing/20131112_279/2013
MK212535|A/canine/Beijing/20131203_342/2013
MK212543|A/canine/Beijing/20131203_341/2013
KJ917347|A/canine/Guangdong/01/2013
KJ917365|A/canine/Guangdong/03/2013
KJ917386|A/canine/Guangdong/04/2013
KJ917373|A/canine/Guangdong/01/2014
KJ917381|A/canine/Guangdong/02/2014
KJ917355|A/canine/Guangdong/02/2013
JN247617|A/canine/Jiangsu/06/2010
JN247577|A/canine/Jiangsu/01/2009
JN247585|A/canine/Jiangsu/02/2010
JN247609|A/canine/Jiangsu/05/2010
JN247601|A/canine/Jiangsu/04/2010
JN247593|A/canine/Jiangsu/03/2010
JX414250|A/canine/Guangdong/05/2011
GU433359|A/canine/Guangdong/2/2006
GU433375|A/canine/Guangdong/1/2007
JX195354|A/canine/Guangdong/2/2011
JX195362|A/canine/Guangdong/3/2011
GU433350|A/canine/Guangdong/1/2006
2005
2007.5
2010
2012.5
2015
2017.5
2020

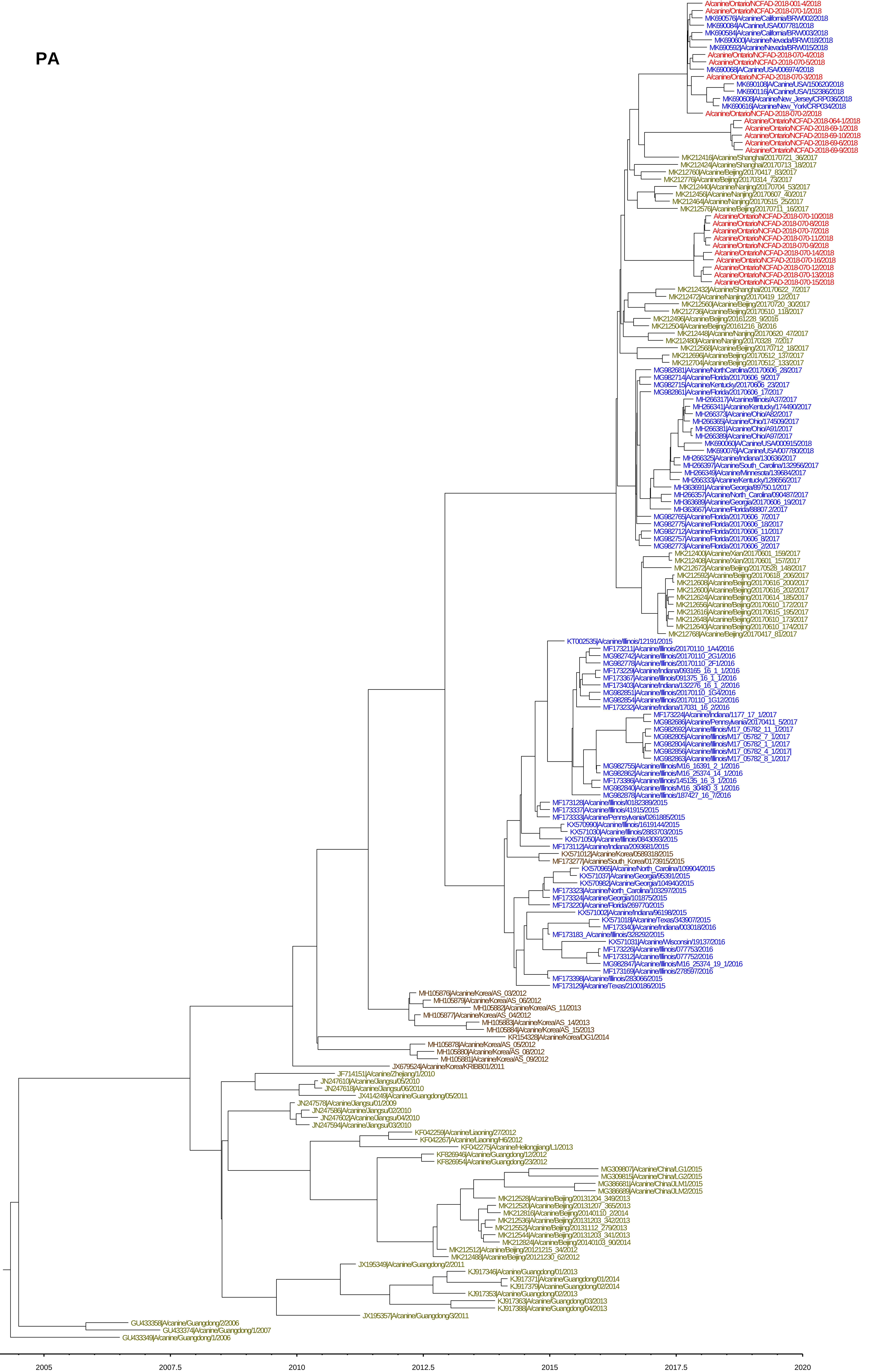
PA
A/canine/Ontario/NCFAD-2018-001-4/2018
A/canine/Ontario/NCFAD-2018-070-1/2018
MK690576|A/canine/California/BRW002/2018
MK690084|A/canine/USA/007781/2018
MK690584|A/canine/California/BRW003/2018
MK690600|A/canine/Nevada/BRW018/2018
MK690592|A/canine/Nevada/BRW015/2018
A/canine/Ontario/NCFAD-2018-070-4/2018
A/canine/Ontario/NCFAD-2018-070-5/2018
MK690068|A/canine/USA/006974/2018
A/canine/Ontario/NCFAD-2018-070-3/2018
MK690108|A/Canine/USA/150620/2018
MK690116|A/Canine/USA/152386/2018
MK690608|A/canine/New_Jersey/CRP036/2018
MK690616|A/canine/New_York/CRP034/2018
A/canine/Ontario/NCFAD-2018-070-2/2018
A/canine/Ontario/NCFAD-2018-064-1/2018
A/canine/Ontario/NCFAD-2018-69-1/2018
A/canine/Ontario/NCFAD-2018-69-10/2018
A/canine/Ontario/NCFAD-2018-69-6/2018
A/canine/Ontario/NCFAD-2018-69-9/2018
MK212416|A/canine/Shanghai/20170721_36/2017
MK212424|A/canine/Shanghai/20170713_18/2017
MK212760|A/canine/Beijing/20170417_83/2017
MK212776|A/canine/Beijing/20170314_73/2017
MK212440|A/canine/Nanjing/20170704_53/2017
MK212456|A/canine/Nanjing/20170607_40/2017
MK212464|A/canine/Nanjing/20170515_25/2017
MK212576|A/canine/Beijing/20170711_16/2017
A/canine/Ontario/NCFAD-2018-070-10/2018
A/canine/Ontario/NCFAD-2018-070-8/2018
A/canine/Ontario/NCFAD-2018-070-7/2018
A/canine/Ontario/NCFAD-2018-070-11/2018
A/canine/Ontario/NCFAD-2018-070-9/2018
A/canine/Ontario/NCFAD-2018-070-14/2018
A/canine/Ontario/NCFAD-2018-070-16/2018
A/canine/Ontario/NCFAD-2018-070-12/2018
A/canine/Ontario/NCFAD-2018-070-13/2018
A/canine/Ontario/NCFAD-2018-070-15/2018
MK212432|A/canine/Shanghai/20170622_7/2017
MK212472|A/canine/Nanjing/20170419_12/2017
MK212560|A/canine/Beijing/20170720_30/2017
MK212736|A/canine/Beijing/20170510_118/2017
MK212496|A/canine/Beijing/20161228_9/2016
MK212504|A/canine/Beijing/20161216_8/2016
MK212448|A/canine/Nanjing/20170620_47/2017
MK212480|A/canine/Nanjing/20170328_7/2017
MK212568|A/canine/Beijing/20170712_18/2017
MK212696|A/canine/Beijing/20170512_137/2017
MK212704|A/canine/Beijing/20170512_133/2017
MG982681|A/canine/NorthCarolina/20170606_28/2017
MG982714|A/canine/Florida/20170606_9/2017
MG982715|A/canine/Kentucky/20170606_23/2017
MG982861|A/canine/Florida/20170606_17/2017
MH266317|A/canine/Illinois/A37/2017
MH266341|A/canine/Kentucky/174490/2017
MH266373|A/canine/Ohio/A82/2017
MH266365|A/canine/Ohio/174509/2017
MH266381|A/canine/Ohio/A91/2017
MH266389|A/canine/Ohio/A97/2017
MK690060|A/Canine/USA/000915/2018
MK690076|A/Canine/USA/007780/2018
MH266325|A/canine/Indiana/130636/2017
MH266397|A/canine/South_Carolina/132956/2017
MH266349|A/canine/Minnesota/139684/2017
MH266333|A/canine/Kentucky/128656/2017
MH363691|A/canine/Georgia/89750.1/2017
MH266357|A/canine/North_Carolina/090487/2017
MH363689|A/canine/Georgia/20170606_19/2017
MH363667|A/canine/Florida/88807.2/2017
MG982765|A/canine/Florida/20170606_7/2017
MG982775|A/canine/Florida/20170606_18/2017
MG982712|A/canine/Florida/20170606_11/2017
MG982757|A/canine/Florida/20170606_8/2017
MG982773|A/canine/Florida/20170606_2/2017
MK212400|A/canine/Xian/20170601_159/2017
MK212408|A/canine/Xian/20170601_157/2017
MK212672|A/canine/Beijing/20170528_148/2017
MK212592|A/canine/Beijing/20170618_206/2017
MK212608|A/canine/Beijing/20170616_200/2017
MK212600|A/canine/Beijing/20170616_202/2017
MK212624|A/canine/Beijing/20170614_185/2017
MK212656|A/canine/Beijing/20170610_172/2017
MK212616|A/canine/Beijing/20170615_195/2017
MK212648|A/canine/Beijing/20170610_173/2017
MK212640|A/canine/Beijing/20170610_174/2017
MK212768|A/canine/Beijing/20170417_81/2017
KT002535|A/canine/Illinois/12191/2015
MF173211|A/canine/Illinois/20170110_1A4/2016
MG982742|A/canine/Illinois/20170110_2G1/2016
MG982778|A/canine/Illinois/20170110_2F1/2016
MF173229|A/canine/Indiana/093165_16_1_1/2016
MF173367|A/canine/Illinois/091375_16_1_1/2016
MF173403|A/canine/Indiana/132276_16_1_2/2016
MG982851|A/canine/Illinois/20170110_1G4/2016
MG982854|A/canine/Illinois/20170110_1G12/2016
MF173232|A/canine/Indiana/17031_16_2/2016
MF173224|A/canine/Indiana/1177_17_1/2017
MG982686|A/canine/Pennsylvania/20170411_5/2017
MG982692|A/canine/Illinois/M17_05782_11_1/2017
MG982805|A/canine/Illinois/M17_05782_7_1/2017
MG982804|A/canine/Illinois/M17_05782_1_1/2017
MG982856|A/canine/Illinois/M17_05782_4_1/2017
MG982863|A/canine/Illinois/M17_05782_8_1/2017
MG982755|A/canine/Illinois/M16_16391_2_1/2016
MG982862|A/canine/Illinois/M16_25374_14_1/2016
MF173386|A/canine/Illinois/145135_16_3_1/2016
MG982840|A/canine/Illinois/M16_30480_3_1/2016
MG982878|A/canine/Illinois/187427_16_7/2016
MF173128|A/canine/Illinois/I0182389/2015
MF173337|A/canine/Illinois/41915/2015
MF173333|A/canine/Pennsylvania/0261885/2015
KX570990|A/canine/Illinois/1619144/2015
KX571030|A/canine/Illinois/2883703/2015
KX571050|A/canine/Illinois/0843093/2015
MF173112|A/canine/Indiana/2093681/2015
KX571012|A/canine/Korea/0589318/2015
MF173277|A/canine/South_Korea/0173915/2015
KX570965|A/canine/North_Carolina/109904/2015
KX571037|A/canine/Georgia/95391/2015
KX570982|A/canine/Georgia/104940/2015
MF173323|A/canine/North_Carolina/103297/2015
MF173324|A/canine/Georgia/101875/2015
MF173220|A/canine/Florida/269770/2015
KX571002|A/canine/Indiana/96198/2015
KX571018|A/canine/Texas/343907/2015
MF173340|A/canine/Indiana/003018/2016
MF173183_A/canine/Illinois/328292/2015
KX571031|A/canine/Wisconsin/19137/2016
MF173226|A/canine/Illinois/077753/2016
MF173312|A/canine/Illinois/077752/2016
MG982847|A/canine/Illinois/M16_25374_19_1/2016
MF173169|A/canine/Illinois/278597/2016
MF173398|A/canine/Illinois/283066/2015
MF173129|A/canine/Texas/2100186/2015
MH105876|A/canine/Korea/AS_03/2012
MH105879|A/canine/Korea/AS_06/2012
MH105882|A/canine/Korea/AS_11/2013
MH105877|A/canine/Korea/AS_04/2012
MH105883|A/canine/Korea/AS_14/2013
MH105884|A/canine/Korea/AS_15/2013
KR154328|A/canine/Korea/DG1/2014
MH105878|A/canine/Korea/AS_05/2012
MH105880|A/canine/Korea/AS_08/2012
MH105881|A/canine/Korea/AS_09/2012
JX679524|A/canine/Korea/KRIBB01/2011
JF714151|A/canine/Zhejiang/1/2010
JN247610|A/canine/Jiangsu/05/2010
JN247618|A/canine/Jiangsu/06/2010
JX414249|A/canine/Guangdong/05/2011
JN247578|A/canine/Jiangsu/01/2009
JN247586|A/canine/Jiangsu/02/2010
JN247602|A/canine/Jiangsu/04/2010
JN247594|A/canine/Jiangsu/03/2010
KF042259|A/canine/Liaoning/27/2012
KF042267|A/canine/Liaoning/H6/2012
KF042275|A/canine/Heilongjiang/L1/2013
KF826946|A/canine/Guangdong/12/2012
KF826954|A/canine/Guangdong/23/2012
MG309807|A/canine/China/LG1/2015
MG309815|A/canine/China/LG2/2015
MG386681|A/canine/China/JLM1/2015
MG386689|A/canine/China/JLM2/2015
MK212528|A/canine/Beijing/20131204_349/2013
MK212520|A/canine/Beijing/20131207_365/2013
MK212816|A/canine/Beijing/20140110_2/2014
MK212536|A/canine/Beijing/20131203_342/2013
MK212552|A/canine/Beijing/20131112_279/2013
MK212544|A/canine/Beijing/20131203_341/2013
MK212824|A/canine/Beijing/20140103_90/2014
MK212512|A/canine/Beijing/20121215_34/2012
MK212488|A/canine/Beijing/20121230_62/2012
JX195349|A/canine/Guangdong/2/2011
KJ917346|A/canine/Guangdong/01/2013
KJ917371|A/canine/Guangdong/01/2014
KJ917379|A/canine/Guangdong/02/2014
KJ917353|A/canine/Guangdong/02/2013
KJ917363|A/canine/Guangdong/03/2013
KJ917388|A/canine/Guangdong/04/2013
JX195357|A/canine/Guangdong/3/2011
GU433358|A/canine/Guangdong/2/2006
GU433374|A/canine/Guangdong/1/2007
GU433349|A/canine/Guangdong/1/2006
2005
2007.5
2010
2012.5
2015
2017.5
2020

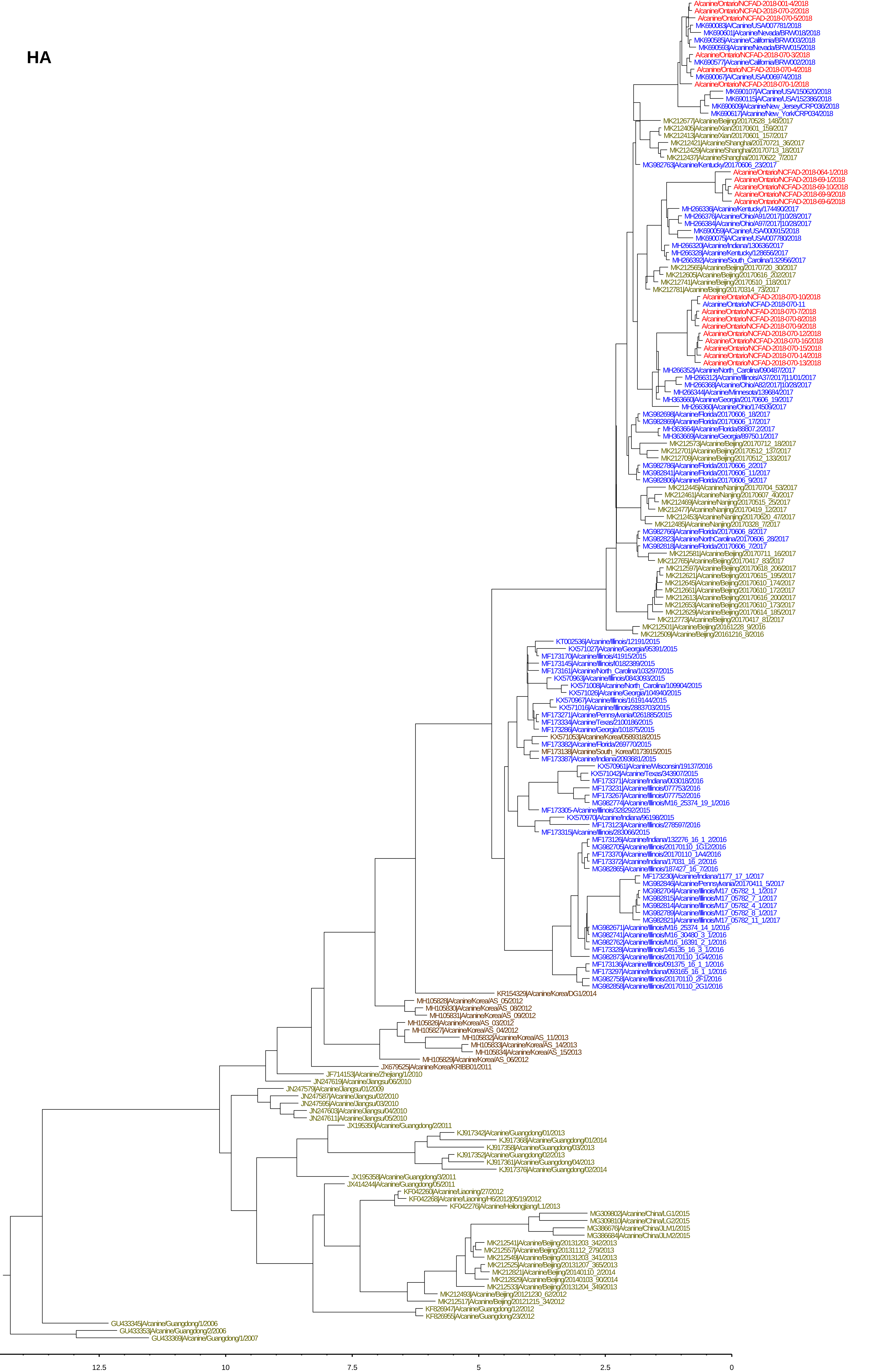

HA
A/canine/Ontario/NCFAD-2018-001-4/2018
A/canine/Ontario/NCFAD-2018-070-2/2018
A/canine/Ontario/NCFAD-2018-070-5/2018
MK690083|A/Canine/USA/007781/2018
MK690601|A/canine/Nevada/BRW018/2018
MK690585|A/canine/California/BRW003/2018
MK690593|A/canine/Nevada/BRW015/2018
A/canine/Ontario/NCFAD-2018-070-3/2018
MK690577|A/canine/California/BRW002/2018
A/canine/Ontario/NCFAD-2018-070-4/2018
MK690067|A/Canine/USA/006974/2018
A/canine/Ontario/NCFAD-2018-070-1/2018
MK690107|A/Canine/USA/150620/2018
MK690115|A/Canine/USA/152386/2018
MK690609|A/canine/New_Jersey/CRP036/2018
MK690617|A/canine/New_York/CRP034/2018
MK212677|A/canine/Beijing/20170528_148/2017
MK212405|A/canine/Xian/20170601_159/2017
MK212413|A/canine/Xian/20170601_157/2017
MK212421|A/canine/Shanghai/20170721_36/2017
MK212429|A/canine/Shanghai/20170713_18/2017
MK212437|A/canine/Shanghai/20170622_7/2017
MG982763|A/canine/Kentucky/20170606_23/2017
A/canine/Ontario/NCFAD-2018-064-1/2018
A/canine/Ontario/NCFAD-2018-69-1/2018
A/canine/Ontario/NCFAD-2018-69-10/2018
A/canine/Ontario/NCFAD-2018-69-9/2018
A/canine/Ontario/NCFAD-2018-69-6/2018
MH266336|A/canine/Kentucky/174490/2017
MH266376|A/canine/Ohio/A91/2017|10/28/2017
MH266384|A/canine/Ohio/A97/2017|10/28/2017
MK690059|A/Canine/USA/000915/2018
MK690075|A/Canine/USA/007780/2018
MH266320|A/canine/Indiana/130636/2017
MH266328|A/canine/Kentucky/128656/2017
MH266392|A/canine/South_Carolina/132956/2017
MK212565|A/canine/Beijing/20170720_30/2017
MK212605|A/canine/Beijing/20170616_202/2017
MK212741|A/canine/Beijing/20170510_118/2017
MK212781|A/canine/Beijing/20170314_73/2017
A/canine/Ontario/NCFAD-2018-070-10/2018
A/canine/Ontario/NCFAD-2018-070-11
A/canine/Ontario/NCFAD-2018-070-7/2018
A/canine/Ontario/NCFAD-2018-070-8/2018
A/canine/Ontario/NCFAD-2018-070-9/2018
A/canine/Ontario/NCFAD-2018-070-12/2018
A/canine/Ontario/NCFAD-2018-070-16/2018
A/canine/Ontario/NCFAD-2018-070-15/2018
A/canine/Ontario/NCFAD-2018-070-14/2018
A/canine/Ontario/NCFAD-2018-070-13/2018
MH266352|A/canine/North_Carolina/090487/2017
MH266312|A/canine/Illinois/A37/2017|11/01/2017
MH266368|A/canine/Ohio/A82/2017|10/28/2017
MH266344|A/canine/Minnesota/139684/2017
MH363660|A/canine/Georgia/20170606_19/2017
MH266360|A/canine/Ohio/174509/2017
MG982698|A/canine/Florida/20170606_18/2017
MG982869|A/canine/Florida/20170606_17/2017
MH363664|A/canine/Florida/88807.2/2017
MH363669|A/canine/Georgia/89750.1/2017
MK212573|A/canine/Beijing/20170712_18/2017
MK212701|A/canine/Beijing/20170512_137/2017
MK212709|A/canine/Beijing/20170512_133/2017
MG982786|A/canine/Florida/20170606_2/2017
MG982841|A/canine/Florida/20170606_11/2017
MG982806|A/canine/Florida/20170606_9/2017
MK212445|A/canine/Nanjing/20170704_53/2017
MK212461|A/canine/Nanjing/20170607_40/2017
MK212469|A/canine/Nanjing/20170515_25/2017
MK212477|A/canine/Nanjing/20170419_12/2017
MK212453|A/canine/Nanjing/20170620_47/2017
MK212485|A/canine/Nanjing/20170328_7/2017
MG982766|A/canine/Florida/20170606_8/2017
MG982823|A/canine/NorthCarolina/20170606_28/2017
MG982818|A/canine/Florida/20170606_7/2017
MK212581|A/canine/Beijing/20170711_16/2017
MK212765|A/canine/Beijing/20170417_83/2017
MK212597|A/canine/Beijing/20170618_206/2017
MK212621|A/canine/Beijing/20170615_195/2017
MK212645|A/canine/Beijing/20170610_174/2017
MK212661|A/canine/Beijing/20170610_172/2017
MK212613|A/canine/Beijing/20170616_200/2017
MK212653|A/canine/Beijing/20170610_173/2017
MK212629|A/canine/Beijing/20170614_185/2017
MK212773|A/canine/Beijing/20170417_81/2017
MK212501|A/canine/Beijing/20161228_9/2016
MK212509|A/canine/Beijing/20161216_8/2016
KT002536|A/canine/Illinois/12191/2015
KX571027|A/canine/Georgia/95391/2015
MF173170|A/canine/Illinois/41915/2015
MF173145|A/canine/Illinois/I0182389/2015
MF173161|A/canine/North_Carolina/103297/2015
KX570963|A/canine/Illinois/0843093/2015
KX571008|A/canine/North_Carolina/109904/2015
KX571026|A/canine/Georgia/104940/2015
KX570967|A/canine/Illinois/1619144/2015
KX571016|A/canine/Illinois/2883703/2015
MF173271|A/canine/Pennsylvania/0261885/2015
MF173334|A/canine/Texas/2100186/2015
MF173286|A/canine/Georgia/101875/2015
KX571053|A/canine/Korea/0589318/2015
MF173382|A/canine/Florida/269770/2015
MF173138|A/canine/South_Korea/0173915/2015
MF173387|A/canine/Indiana/2093681/2015
KX570961|A/canine/Wisconsin/19137/2016
KX571042|A/canine/Texas/343907/2015
MF173371|A/canine/Indiana/003018/2016
MF173231|A/canine/Illinois/077753/2016
MF173267|A/canine/Illinois/077752/2016
MG982774|A/canine/Illinois/M16_25374_19_1/2016
MF173305-A/canine/Illinois/328292/2015
KX570970|A/canine/Indiana/96198/2015
MF173123|A/canine/Illinois/278597/2016
MF173315|A/canine/Illinois/283066/2015
MF173126|A/canine/Indiana/132276_16_1_2/2016
MG982705|A/canine/Illinois/20170110_1G12/2016
MF173370|A/canine/Illinois/20170110_1A4/2016
MF173372|A/canine/Indiana/17031_16_2/2016
MG982865|A/canine/Illinois/187427_16_7/2016
MF173230|A/canine/Indiana/1177_17_1/2017
MG982846|A/canine/Pennsylvania/20170411_5/2017
MG982704|A/canine/Illinois/M17_05782_1_1/2017
MG982815|A/canine/Illinois/M17_05782_7_1/2017
MG982814|A/canine/Illinois/M17_05782_4_1/2017
MG982789|A/canine/Illinois/M17_05782_8_1/2017
MG982821|A/canine/Illinois/M17_05782_11_1/2017
MG982671|A/canine/Illinois/M16_25374_14_1/2016
MG982741|A/canine/Illinois/M16_30480_3_1/2016
MG982762|A/canine/Illinois/M16_16391_2_1/2016
MF173328|A/canine/Illinois/145135_16_3_1/2016
MG982873|A/canine/Illinois/20170110_1G4/2016
MF173136|A/canine/Illinois/091375_16_1_1/2016
MF173297|A/canine/Indiana/093165_16_1_1/2016
MG982758|A/canine/Illinois/20170110_2F1/2016
MG982858|A/canine/Illinois/20170110_2G1/2016
KR154329|A/canine/Korea/DG1/2014
MH105828|A/canine/Korea/AS_05/2012
MH105830|A/canine/Korea/AS_08/2012
MH105831|A/canine/Korea/AS_09/2012
MH105826|A/canine/Korea/AS_03/2012
MH105827|A/canine/Korea/AS_04/2012
MH105832|A/canine/Korea/AS_11/2013
MH105833|A/canine/Korea/AS_14/2013
MH105834|A/canine/Korea/AS_15/2013
MH105829|A/canine/Korea/AS_06/2012
JX679525|A/canine/Korea/KRIBB01/2011
JF714153|A/canine/Zhejiang/1/2010
JN247619|A/canine/Jiangsu/06/2010
JN247579|A/canine/Jiangsu/01/2009
JN247587|A/canine/Jiangsu/02/2010
JN247595|A/canine/Jiangsu/03/2010
JN247603|A/canine/Jiangsu/04/2010
JN247611|A/canine/Jiangsu/05/2010
JX195350|A/canine/Guangdong/2/2011
KJ917342|A/canine/Guangdong/01/2013
KJ917368|A/canine/Guangdong/01/2014
KJ917358|A/canine/Guangdong/03/2013
KJ917352|A/canine/Guangdong/02/2013
KJ917361|A/canine/Guangdong/04/2013
KJ917376|A/canine/Guangdong/02/2014
JX195358|A/canine/Guangdong/3/2011
JX414244|A/canine/Guangdong/05/2011
KF042260|A/canine/Liaoning/27/2012
KF042268|A/canine/Liaoning/H6/2012|05/19/2012
KF042276|A/canine/Heilongjiang/L1/2013
MG309802|A/canine/China/LG1/2015
MG309810|A/canine/China/LG2/2015
MG386676|A/canine/China/JLM1/2015
MG386684|A/canine/China/JLM2/2015
MK212541|A/canine/Beijing/20131203_342/2013
MK212557|A/canine/Beijing/20131112_279/2013
MK212549|A/canine/Beijing/20131203_341/2013
MK212525|A/canine/Beijing/20131207_365/2013
MK212821|A/canine/Beijing/20140110_2/2014
MK212829|A/canine/Beijing/20140103_90/2014
MK212533|A/canine/Beijing/20131204_349/2013
MK212493|A/canine/Beijing/20121230_62/2012
MK212517|A/canine/Beijing/20121215_34/2012
KF826947|A/canine/Guangdong/12/2012
KF826955|A/canine/Guangdong/23/2012
GU433345|A/canine/Guangdong/1/2006
GU433353|A/canine/Guangdong/2/2006
GU433369|A/canine/Guangdong/1/2007
12.5
10
7.5
5
2.5
0

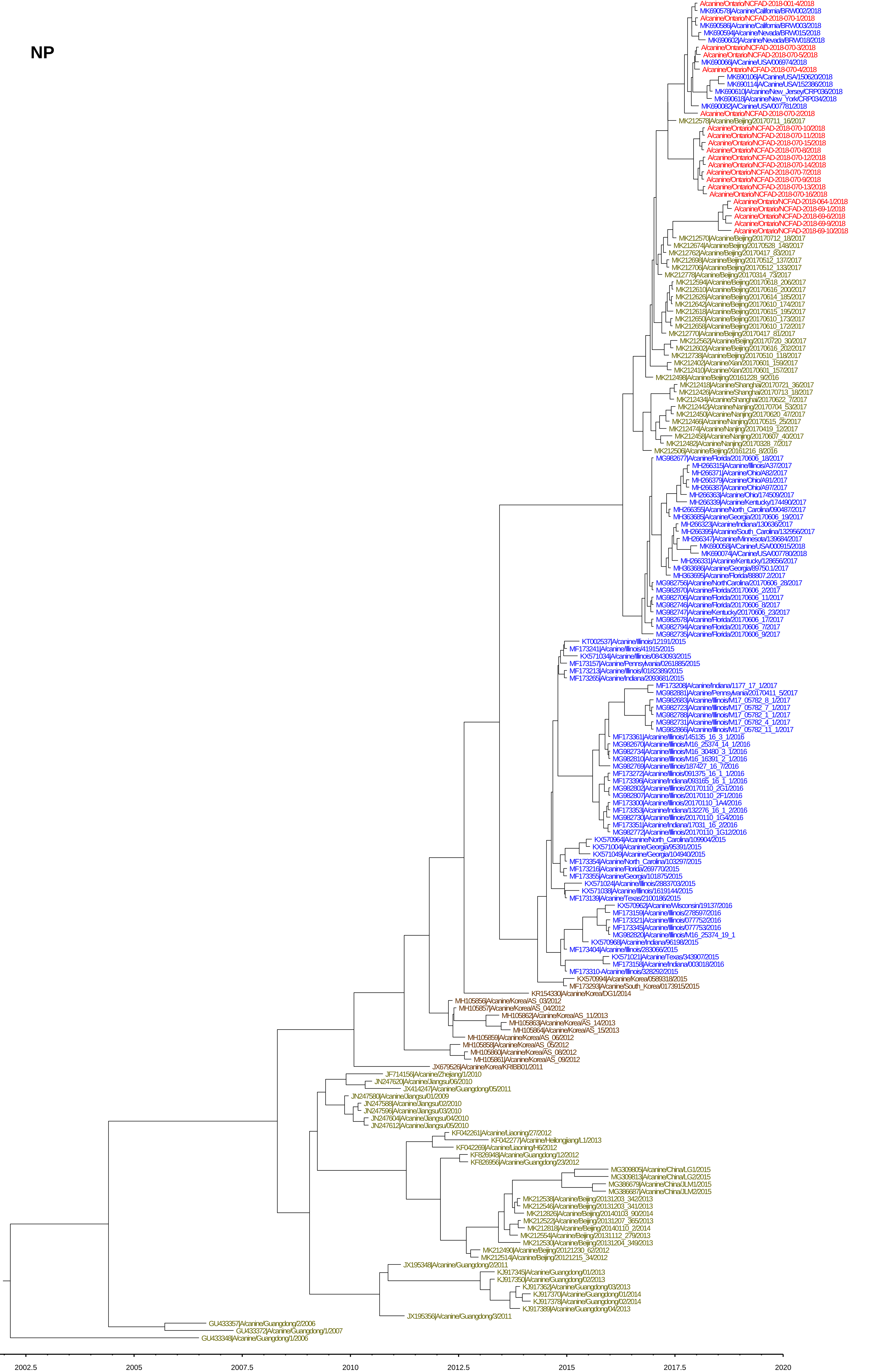
NP
A/canine/Ontario/NCFAD-2018-001-4/2018
MK690578|A/canine/California/BRW002/2018
A/canine/Ontario/NCFAD-2018-070-1/2018
MK690586|A/canine/California/BRW003/2018
MK690594|A/canine/Nevada/BRW015/2018
MK690602|A/canine/Nevada/BRW018/2018
A/canine/Ontario/NCFAD-2018-070-3/2018
A/canine/Ontario/NCFAD-2018-070-5/2018
MK690066|A/Canine/USA/006974/2018
A/canine/Ontario/NCFAD-2018-070-4/2018
MK690106|A/Canine/USA/150620/2018
MK690114|A/Canine/USA/152386/2018
MK690610|A/canine/New_Jersey/CRP036/2018
MK690618|A/canine/New_York/CRP034/2018
MK690082|A/Canine/USA/007781/2018
A/canine/Ontario/NCFAD-2018-070-2/2018
MK212578|A/canine/Beijing/20170711_16/2017
A/canine/Ontario/NCFAD-2018-070-10/2018
A/canine/Ontario/NCFAD-2018-070-11/2018
A/canine/Ontario/NCFAD-2018-070-15/2018
A/canine/Ontario/NCFAD-2018-070-8/2018
A/canine/Ontario/NCFAD-2018-070-12/2018
A/canine/Ontario/NCFAD-2018-070-14/2018
A/canine/Ontario/NCFAD-2018-070-7/2018
A/canine/Ontario/NCFAD-2018-070-9/2018
A/canine/Ontario/NCFAD-2018-070-13/2018
A/canine/Ontario/NCFAD-2018-070-16/2018
A/canine/Ontario/NCFAD-2018-064-1/2018
A/canine/Ontario/NCFAD-2018-69-1/2018
A/canine/Ontario/NCFAD-2018-69-6/2018
A/canine/Ontario/NCFAD-2018-69-9/2018
A/canine/Ontario/NCFAD-2018-69-10/2018
MK212570|A/canine/Beijing/20170712_18/2017
MK212674|A/canine/Beijing/20170528_148/2017
MK212762|A/canine/Beijing/20170417_83/2017
MK212698|A/canine/Beijing/20170512_137/2017
MK212706|A/canine/Beijing/20170512_133/2017
MK212778|A/canine/Beijing/20170314_73/2017
MK212594|A/canine/Beijing/20170618_206/2017
MK212610|A/canine/Beijing/20170616_200/2017
MK212626|A/canine/Beijing/20170614_185/2017
MK212642|A/canine/Beijing/20170610_174/2017
MK212618|A/canine/Beijing/20170615_195/2017
MK212650|A/canine/Beijing/20170610_173/2017
MK212658|A/canine/Beijing/20170610_172/2017
MK212770|A/canine/Beijing/20170417_81/2017
MK212562|A/canine/Beijing/20170720_30/2017
MK212602|A/canine/Beijing/20170616_202/2017
MK212738|A/canine/Beijing/20170510_118/2017
MK212402|A/canine/Xian/20170601_159/2017
MK212410|A/canine/Xian/20170601_157/2017
MK212498|A/canine/Beijing/20161228_9/2016
MK212418|A/canine/Shanghai/20170721_36/2017
MK212426|A/canine/Shanghai/20170713_18/2017
MK212434|A/canine/Shanghai/20170622_7/2017
MK212442|A/canine/Nanjing/20170704_53/2017
MK212450|A/canine/Nanjing/20170620_47/2017
MK212466|A/canine/Nanjing/20170515_25/2017
MK212474|A/canine/Nanjing/20170419_12/2017
MK212458|A/canine/Nanjing/20170607_40/2017
MK212482|A/canine/Nanjing/20170328_7/2017
MK212506|A/canine/Beijing/20161216_8/2016
MG982677|A/canine/Florida/20170606_18/2017
MH266315|A/canine/Illinois/A37/2017
MH266371|A/canine/Ohio/A82/2017
MH266379|A/canine/Ohio/A91/2017
MH266387|A/canine/Ohio/A97/2017
MH266363|A/canine/Ohio/174509/2017
MH266339|A/canine/Kentucky/174490/2017
MH266355|A/canine/North_Carolina/090487/2017
MH363685|A/canine/Georgia/20170606_19/2017
MH266323|A/canine/Indiana/130636/2017
MH266395|A/canine/South_Carolina/132956/2017
MH266347|A/canine/Minnesota/139684/2017
MK690058|A/Canine/USA/000915/2018
MK690074|A/Canine/USA/007780/2018
MH266331|A/canine/Kentucky/128656/2017
MH363686|A/canine/Georgia/89750.1/2017
MH363695|A/canine/Florida/88807.2/2017
MG982756|A/canine/NorthCarolina/20170606_28/2017
MG982870|A/canine/Florida/20170606_2/2017
MG982706|A/canine/Florida/20170606_11/2017
MG982746|A/canine/Florida/20170606_8/2017
MG982747|A/canine/Kentucky/20170606_23/2017
MG982678|A/canine/Florida/20170606_17/2017
MG982794|A/canine/Florida/20170606_7/2017
MG982735|A/canine/Florida/20170606_9/2017
KT002537|A/canine/Illinois/12191/2015
MF173241|A/canine/Illinois/41915/2015
KX571034|A/canine/Illinois/0843093/2015
MF173157|A/canine/Pennsylvania/0261885/2015
MF173213|A/canine/Illinois/I0182389/2015
MF173265|A/canine/Indiana/2093681/2015
MF173206|A/canine/Indiana/1177_17_1/2017
MG982681|A/canine/Pennsylvania/20170411_5/2017
MG982683|A/canine/Illinois/M17_05782_8_1/2017
MG982723|A/canine/Illinois/M17_05782_7_1/2017
MG982788|A/canine/Illinois/M17_05782_1_1/2017
MG982731|A/canine/Illinois/M17_05782_4_1/2017
MG982866|A/canine/Illinois/M17_05782_11_1/2017
MF173361|A/canine/Illinois/145135_16_3_1/2016
MG982670|A/canine/Illinois/M16_25374_14_1/2016
MG982734|A/canine/Illinois/M16_30480_3_1/2016
MG982810|A/canine/Illinois/M16_16391_2_1/2016
MG982769|A/canine/Illinois/187427_16_7/2016
MF173272|A/canine/Illinois/091375_16_1_1/2016
MF173396|A/canine/Indiana/093165_16_1_1/2016
MG982802|A/canine/Illinois/20170110_2G1/2016
MG982807|A/canine/Illinois/20170110_2F1/2016
MF173300|A/canine/Illinois/20170110_1A4/2016
MF173353|A/canine/Indiana/132276_16_1_2/2016
MG982730|A/canine/Illinois/20170110_1G4/2016
MF173351|A/canine/Indiana/17031_16_2/2016
MG982772|A/canine/Illinois/20170110_1G12/2016
KX570964|A/canine/North_Carolina/109904/2015
KX571004|A/canine/Georgia/95391/2015
KX571049|A/canine/Georgia/104940/2015
MF173354|A/canine/North_Carolina/103297/2015
MF173216|A/canine/Florida/269770/2015
MF173355|A/canine/Georgia/101875/2015
KX571024|A/canine/Illinois/2883703/2015
KX571038|A/canine/Illinois/1619144/2015
MF173139|A/canine/Texas/2100186/2015
KX570962|A/canine/Wisconsin/19137/2016
MF173159|A/canine/Illinois/278597/2016
MF173321|A/canine/Illinois/077752/2016
MF173345|A/canine/Illinois/077753/2016
MG982820|A/canine/Illinois/M16_25374_19_1
KX570968|A/canine/Indiana/96198/2015
MF173404|A/canine/Illinois/283066/2015
KX571021|A/canine/Texas/343907/2015
MF173158|A/canine/Indiana/003018/2016
MF173310-A/canine/Illinois/328292/2015
KX570994|A/canine/Korea/0589318/2015
MF173293|A/canine/South_Korea/0173915/2015
KR154330|A/canine/Korea/DG1/2014
MH105856|A/canine/Korea/AS_03/2012
MH105857|A/canine/Korea/AS_04/2012
MH105862|A/canine/Korea/AS_11/2013
MH105863|A/canine/Korea/AS_14/2013
MH105864|A/canine/Korea/AS_15/2013
MH105859|A/canine/Korea/AS_06/2012
MH105858|A/canine/Korea/AS_05/2012
MH105860|A/canine/Korea/AS_08/2012
MH105861|A/canine/Korea/AS_09/2012
JX679526|A/canine/Korea/KRIBB01/2011
JF714156|A/canine/Zhejiang/1/2010
JN247620|A/canine/Jiangsu/06/2010
JX414247|A/canine/Guangdong/05/2011
JN247580|A/canine/Jiangsu/01/2009
JN247588|A/canine/Jiangsu/02/2010
JN247596|A/canine/Jiangsu/03/2010
JN247604|A/canine/Jiangsu/04/2010
JN247612|A/canine/Jiangsu/05/2010
KF042261|A/canine/Liaoning/27/2012
KF042277|A/canine/Heilongjiang/L1/2013
KF042269|A/canine/Liaoning/H6/2012
KF826948|A/canine/Guangdong/12/2012
KF826956|A/canine/Guangdong/23/2012
MG309805|A/canine/China/LG1/2015
MG309813|A/canine/China/LG2/2015
MG386679|A/canine/China/JLM1/2015
MG386687|A/canine/China/JLM2/2015
MK212538|A/canine/Beijing/20131203_342/2013
MK212546|A/canine/Beijing/20131203_341/2013
MK212826|A/canine/Beijing/20140103_90/2014
MK212522|A/canine/Beijing/20131207_365/2013
MK212818|A/canine/Beijing/20140110_2/2014
MK212554|A/canine/Beijing/20131112_279/2013
MK212530|A/canine/Beijing/20131204_349/2013
MK212490|A/canine/Beijing/20121230_62/2012
MK212514|A/canine/Beijing/20121215_34/2012
JX195348|A/canine/Guangdong/2/2011
KJ917345|A/canine/Guangdong/01/2013
KJ917350|A/canine/Guangdong/02/2013
KJ917362|A/canine/Guangdong/03/2013
KJ917370|A/canine/Guangdong/01/2014
KJ917378|A/canine/Guangdong/02/2014
KJ917389|A/canine/Guangdong/04/2013
JX195356|A/canine/Guangdong/3/2011
GU433357|A/canine/Guangdong/2/2006
GU433372|A/canine/Guangdong/1/2007
GU433348|A/canine/Guangdong/1/2006
2002.5
2005
2007.5
2010
2012.5
2015
2017.5
2020

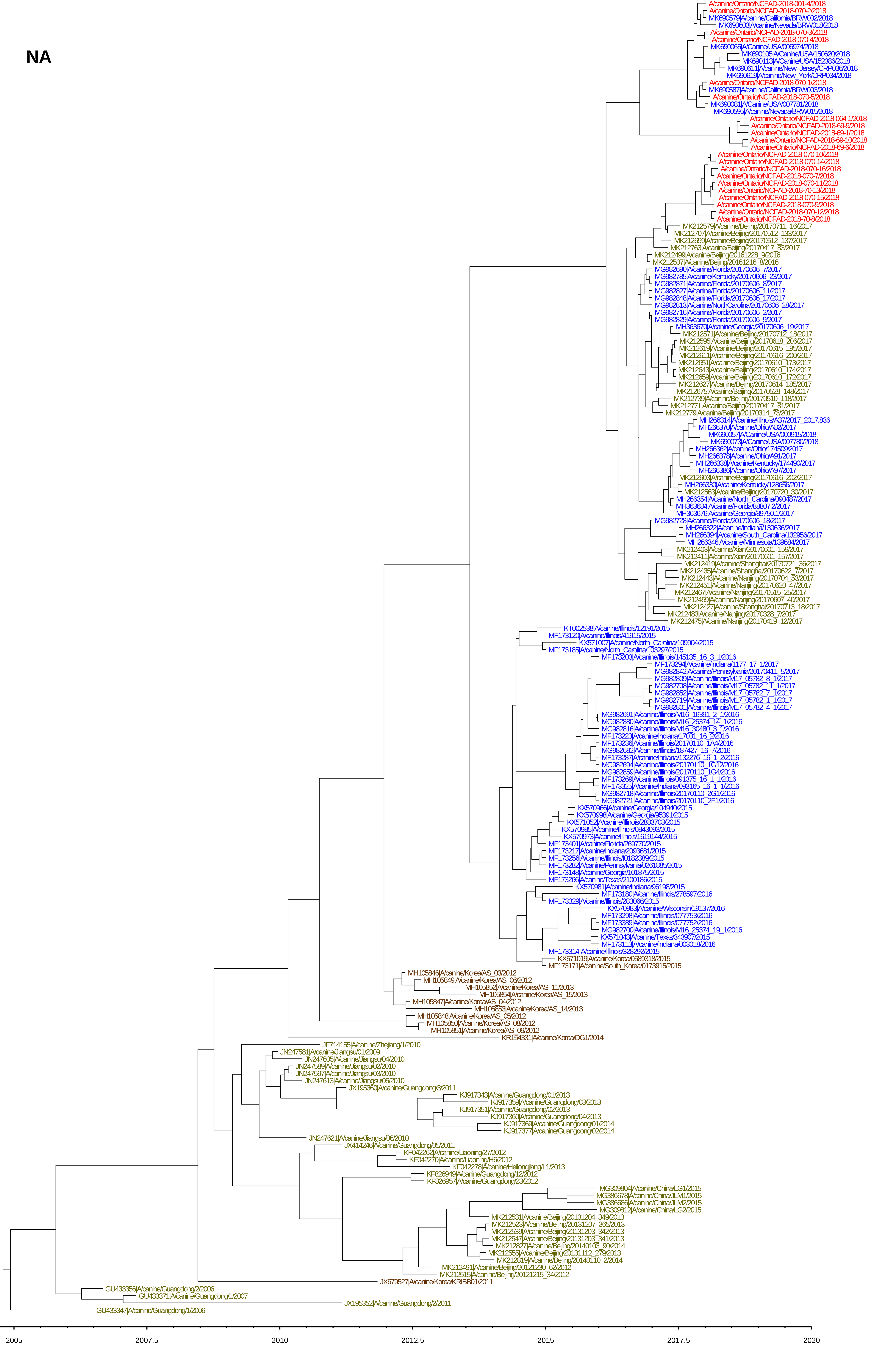
NA
A/canine/Ontario/NCFAD-2018-001-4/2018
A/canine/Ontario/NCFAD-2018-070-2/2018
MK690579|A/canine/California/BRW002/2018
MK690603|A/canine/Nevada/BRW018/2018
A/canine/Ontario/NCFAD-2018-070-3/2018
A/canine/Ontario/NCFAD-2018-070-4/2018
MK690065|A/Canine/USA/006974/2018
MK690105|A/Canine/USA/150620/2018
MK690113|A/Canine/USA/152386/2018
MK690611|A/canine/New_Jersey/CRP036/2018
MK690619|A/canine/New_York/CRP034/2018
A/canine/Ontario/NCFAD-2018-070-1/2018
MK690587|A/canine/California/BRW003/2018
A/canine/Ontario/NCFAD-2018-070-5/2018
MK690081|A/Canine/USA/007781/2018
MK690595|A/canine/Nevada/BRW015/2018
A/canine/Ontario/NCFAD-2018-064-1/2018
A/canine/Ontario/NCFAD-2018-69-9/2018
A/canine/Ontario/NCFAD-2018-69-1/2018
A/canine/Ontario/NCFAD-2018-69-10/2018
A/canine/Ontario/NCFAD-2018-69-6/2018
A/canine/Ontario/NCFAD-2018-070-10/2018
A/canine/Ontario/NCFAD-2018-070-14/2018
A/canine/Ontario/NCFAD-2018-070-16/2018
A/canine/Ontario/NCFAD-2018-070-7/2018
A/canine/Ontario/NCFAD-2018-070-11/2018
A/canine/Ontario/NCFAD-2018-70-13/2018
A/canine/Ontario/NCFAD-2018-070-15/2018
A/canine/Ontario/NCFAD-2018-070-9/2018
A/canine/Ontario/NCFAD-2018-070-12/2018
A/canine/Ontario/NCFAD-2018-70-8/2018
MK212579|A/canine/Beijing/20170711_16/2017
MK212707|A/canine/Beijing/20170512_133/2017
MK212699|A/canine/Beijing/20170512_137/2017
MK212763|A/canine/Beijing/20170417_83/2017
MK212499|A/canine/Beijing/20161228_9/2016
MK212507|A/canine/Beijing/20161216_8/2016
MG982690|A/canine/Florida/20170606_7/2017
MG982785|A/canine/Kentucky/20170606_23/2017
MG982871|A/canine/Florida/20170606_8/2017
MG982827|A/canine/Florida/20170606_11/2017
MG982848|A/canine/Florida/20170606_17/2017
MG982813|A/canine/NorthCarolina/20170606_28/2017
MG982716|A/canine/Florida/20170606_2/2017
MG982829|A/canine/Florida/20170606_9/2017
MH363670|A/canine/Georgia/20170606_19/2017
MK212571|A/canine/Beijing/20170712_18/2017
MK212595|A/canine/Beijing/20170618_206/2017
MK212619|A/canine/Beijing/20170615_195/2017
MK212611|A/canine/Beijing/20170616_200/2017
MK212651|A/canine/Beijing/20170610_173/2017
MK212643|A/canine/Beijing/20170610_174/2017
MK212659|A/canine/Beijing/20170610_172/2017
MK212627|A/canine/Beijing/20170614_185/2017
MK212675|A/canine/Beijing/20170528_148/2017
MK212739|A/canine/Beijing/20170510_118/2017
MK212771|A/canine/Beijing/20170417_81/2017
MK212779|A/canine/Beijing/20170314_73/2017
MH266314|A/canine/Illinois/A37/2017_2017.836
MH266370|A/canine/Ohio/A82/2017
MK690057|A/Canine/USA/000915/2018
MK690073|A/Canine/USA/007780/2018
MH266362|A/canine/Ohio/174509/2017
MH266378|A/canine/Ohio/A91/2017
MH266338|A/canine/Kentucky/174490/2017
MH266386|A/canine/Ohio/A97/2017
MK212603|A/canine/Beijing/20170616_202/2017
MH266330|A/canine/Kentucky/128656/2017
MK212563|A/canine/Beijing/20170720_30/2017
MH266354|A/canine/North_Carolina/090487/2017
MH363684|A/canine/Florida/88807.2/2017
MH363676|A/canine/Georgia/89750.1/2017
MG982728|A/canine/Florida/20170606_18/2017
MH266322|A/canine/Indiana/130636/2017
MH266394|A/canine/South_Carolina/132956/2017
MH266346|A/canine/Minnesota/139684/2017
MK212403|A/canine/Xian/20170601_159/2017
MK212411|A/canine/Xian/20170601_157/2017
MK212419|A/canine/Shanghai/20170721_36/2017
MK212435|A/canine/Shanghai/20170622_7/2017
MK212443|A/canine/Nanjing/20170704_53/2017
MK212451|A/canine/Nanjing/20170620_47/2017
MK212467|A/canine/Nanjing/20170515_25/2017
MK212459|A/canine/Nanjing/20170607_40/2017
MK212427|A/canine/Shanghai/20170713_18/2017
MK212483|A/canine/Nanjing/20170328_7/2017
MK212475|A/canine/Nanjing/20170419_12/2017
KT002538|A/canine/Illinois/12191/2015
MF173120|A/canine/Illinois/41915/2015
KX571007|A/canine/North_Carolina/109904/2015
MF173185|A/canine/North_Carolina/103297/2015
MF173203|A/canine/Illinois/145135_16_3_1/2016
MF173294|A/canine/Indiana/1177_17_1/2017
MG982842|A/canine/Pennsylvania/20170411_5/2017
MG982809|A/canine/Illinois/M17_05782_8_1/2017
MG982708|A/canine/Illinois/M17_05782_11_1/2017
MG982852|A/canine/Illinois/M17_05782_7_1/2017
MG982719|A/canine/Illinois/M17_05782_1_1/2017
MG982801|A/canine/Illinois/M17_05782_4_1/2017
MG982691|A/canine/Illinois/M16_16391_2_1/2016
MG982880|A/canine/Illinois/M16_25374_14_1/2016
MG982816|A/canine/Illinois/M16_30480_3_1/2016
MF173223|A/canine/Indiana/17031_16_2/2016
MF173236|A/canine/Illinois/20170110_1A4/2016
MG982682|A/canine/Illinois/187427_16_7/2016
MF173287|A/canine/Indiana/132276_16_1_2/2016
MG982694|A/canine/Illinois/20170110_1G12/2016
MG982859|A/canine/Illinois/20170110_1G4/2016
MF173269|A/canine/Illinois/091375_16_1_1/2016
MF173325|A/canine/Indiana/093165_16_1_1/2016
MG982718|A/canine/Illinois/20170110_2G1/2016
MG982721|A/canine/Illinois/20170110_2F1/2016
KX570966|A/canine/Georgia/104940/2015
KX570998|A/canine/Georgia/95391/2015
KX571052|A/canine/Illinois/2883703/2015
KX570985|A/canine/Illinois/0843093/2015
KX570973|A/canine/Illinois/1619144/2015
MF173401|A/canine/Florida/269770/2015
MF173217|A/canine/Indiana/2093681/2015
MF173256|A/canine/Illinois/I0182389/2015
MF173282|A/canine/Pennsylvania/0261885/2015
MF173148|A/canine/Georgia/101875/2015
MF173266|A/canine/Texas/2100186/2015
KX570981|A/canine/Indiana/96198/2015
MF173180|A/canine/Illinois/278597/2016
MF173329|A/canine/Illinois/283066/2015
KX570983|A/canine/Wisconsin/19137/2016
MF173298|A/canine/Illinois/077753/2016
MF173389|A/canine/Illinois/077752/2016
MG982700|A/canine/Illinois/M16_25374_19_1/2016
KX571043|A/canine/Texas/343907/2015
MF173113|A/canine/Indiana/003018/2016
MF173314-A/canine/Illinois/328292/2015
KX571019|A/canine/Korea/0589318/2015
MF173171|A/canine/South_Korea/0173915/2015
MH105846|A/canine/Korea/AS_03/2012
MH105849|A/canine/Korea/AS_06/2012
MH105852|A/canine/Korea/AS_11/2013
MH105854|A/canine/Korea/AS_15/2013
MH105847|A/canine/Korea/AS_04/2012
MH105853|A/canine/Korea/AS_14/2013
MH105848|A/canine/Korea/AS_05/2012
MH105850|A/canine/Korea/AS_08/2012
MH105851|A/canine/Korea/AS_09/2012
KR154331|A/canine/Korea/DG1/2014
JF714155|A/canine/Zhejiang/1/2010
JN247581|A/canine/Jiangsu/01/2009
JN247605|A/canine/Jiangsu/04/2010
JN247589|A/canine/Jiangsu/02/2010
JN247597|A/canine/Jiangsu/03/2010
JN247613|A/canine/Jiangsu/05/2010
JX195360|A/canine/Guangdong/3/2011
KJ917343|A/canine/Guangdong/01/2013
KJ917359|A/canine/Guangdong/03/2013
KJ917351|A/canine/Guangdong/02/2013
KJ917360|A/canine/Guangdong/04/2013
KJ917369|A/canine/Guangdong/01/2014
KJ917377|A/canine/Guangdong/02/2014
JN247621|A/canine/Jiangsu/06/2010
JX414246|A/canine/Guangdong/05/2011
KF042262|A/canine/Liaoning/27/2012
KF042270|A/canine/Liaoning/H6/2012
KF042278|A/canine/Heilongjiang/L1/2013
KF826949|A/canine/Guangdong/12/2012
KF826957|A/canine/Guangdong/23/2012
MG309804|A/canine/China/LG1/2015
MG386678|A/canine/China/JLM1/2015
MG386686|A/canine/China/JLM2/2015
MG309812|A/canine/China/LG2/2015
MK212531|A/canine/Beijing/20131204_349/2013
MK212523|A/canine/Beijing/20131207_365/2013
MK212539|A/canine/Beijing/20131203_342/2013
MK212547|A/canine/Beijing/20131203_341/2013
MK212827|A/canine/Beijing/20140103_90/2014
MK212555|A/canine/Beijing/20131112_279/2013
MK212819|A/canine/Beijing/20140110_2/2014
MK212491|A/canine/Beijing/20121230_62/2012
MK212515|A/canine/Beijing/20121215_34/2012
JX679527|A/canine/Korea/KRIBB01/2011
GU433356|A/canine/Guangdong/2/2006
GU433371|A/canine/Guangdong/1/2007
JX195352|A/canine/Guangdong/2/2011
GU433347|A/canine/Guangdong/1/2006
2005
2007.5
2010
2012.5
2015
2017.5
2020

M

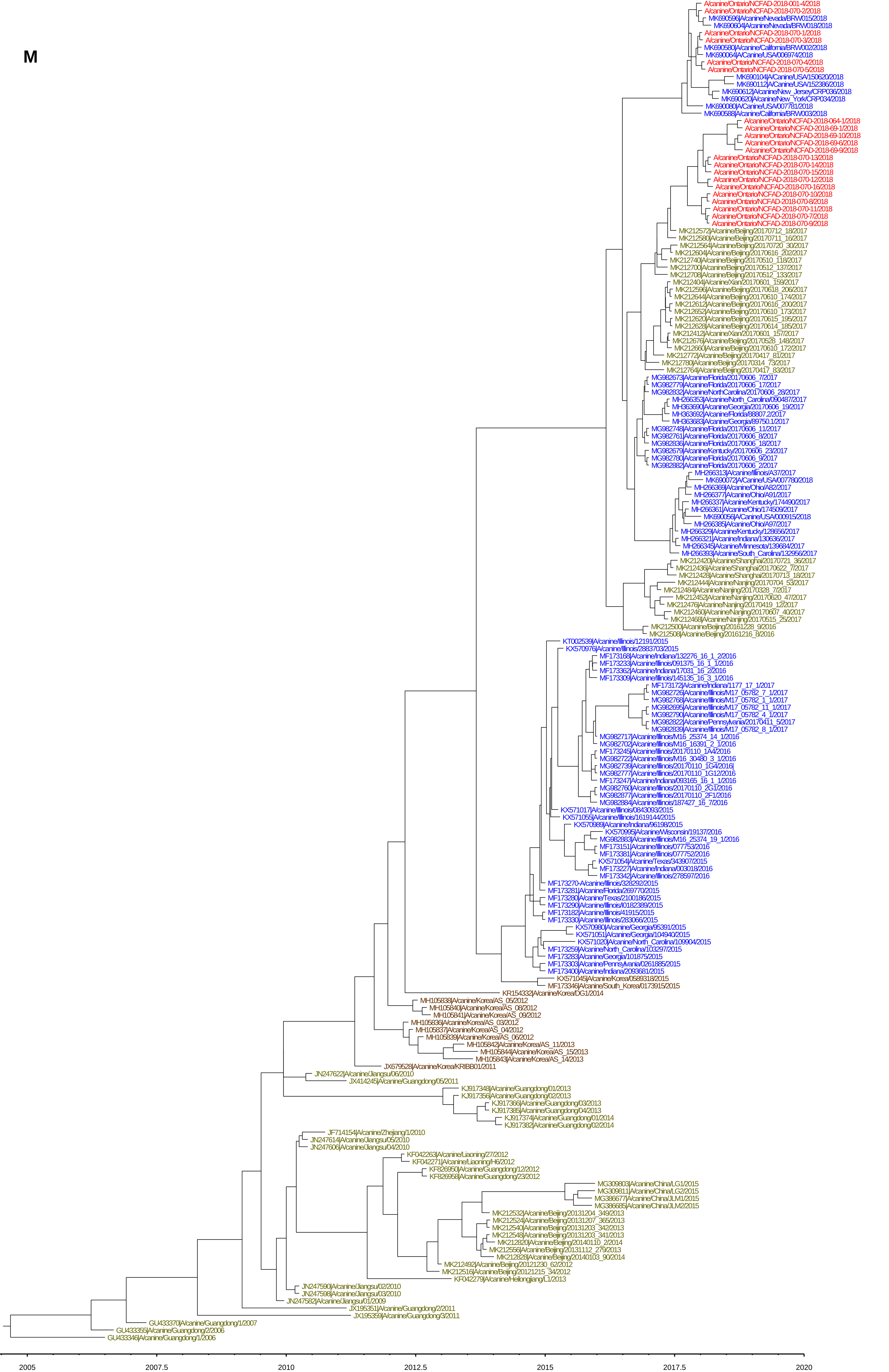

A/canine/Ontario/NCFAD-2018-001-4/2018
A/canine/Ontario/NCFAD-2018-070-2/2018
MK690596|A/canine/Nevada/BRW015/2018
MK690604|A/canine/Nevada/BRW018/2018
A/canine/Ontario/NCFAD-2018-070-1/2018
A/canine/Ontario/NCFAD-2018-070-3/2018
MK690580|A/canine/California/BRW002/2018
MK690064|A/Canine/USA/006974/2018
A/canine/Ontario/NCFAD-2018-070-4/2018
A/canine/Ontario/NCFAD-2018-070-5/2018
MK690104|A/Canine/USA/150620/2018
MK690112|A/Canine/USA/152386/2018
MK690612|A/canine/New_Jersey/CRP036/2018
MK690620|A/canine/New_York/CRP034/2018
MK690080|A/Canine/USA/007781/2018
MK690588|A/canine/California/BRW003/2018
A/canine/Ontario/NCFAD-2018-064-1/2018
A/canine/Ontario/NCFAD-2018-69-1/2018
A/canine/Ontario/NCFAD-2018-69-10/2018
A/canine/Ontario/NCFAD-2018-69-6/2018
A/canine/Ontario/NCFAD-2018-69-9/2018
A/canine/Ontario/NCFAD-2018-070-13/2018
A/canine/Ontario/NCFAD-2018-070-14/2018
A/canine/Ontario/NCFAD-2018-070-15/2018
A/canine/Ontario/NCFAD-2018-070-12/2018
A/canine/Ontario/NCFAD-2018-070-16/2018
A/canine/Ontario/NCFAD-2018-070-10/2018
A/canine/Ontario/NCFAD-2018-070-8/2018
A/canine/Ontario/NCFAD-2018-070-11/2018
A/canine/Ontario/NCFAD-2018-070-7/2018
A/canine/Ontario/NCFAD-2018-070-9/2018
MK212572|A/canine/Beijing/20170712_18/2017
MK212580|A/canine/Beijing/20170711_16/2017
MK212564|A/canine/Beijing/20170720_30/2017
MK212604|A/canine/Beijing/20170616_202/2017
MK212740|A/canine/Beijing/20170510_118/2017
MK212700|A/canine/Beijing/20170512_137/2017
MK212708|A/canine/Beijing/20170512_133/2017
MK212404|A/canine/Xian/20170601_159/2017
MK212596|A/canine/Beijing/20170618_206/2017
MK212644|A/canine/Beijing/20170610_174/2017
MK212612|A/canine/Beijing/20170616_200/2017
MK212652|A/canine/Beijing/20170610_173/2017
MK212620|A/canine/Beijing/20170615_195/2017
MK212628|A/canine/Beijing/20170614_185/2017
MK212412|A/canine/Xian/20170601_157/2017
MK212676|A/canine/Beijing/20170528_148/2017
MK212660|A/canine/Beijing/20170610_172/2017
MK212772|A/canine/Beijing/20170417_81/2017
MK212780|A/canine/Beijing/20170314_73/2017
MK212764|A/canine/Beijing/20170417_83/2017
MG982673|A/canine/Florida/20170606_7/2017
MG982779|A/canine/Florida/20170606_17/2017
MG982832|A/canine/NorthCarolina/20170606_28/2017
MH266353|A/canine/North_Carolina/090487/2017
MH363690|A/canine/Georgia/20170606_19/2017
MH363692|A/canine/Florida/88807.2/2017
MH363683|A/canine/Georgia/89750.1/2017
MG982748|A/canine/Florida/20170606_11/2017
MG982761|A/canine/Florida/20170606_8/2017
MG982836|A/canine/Florida/20170606_18/2017
MG982679|A/canine/Kentucky/20170606_23/2017
MG982780|A/canine/Florida/20170606_9/2017
MG982882|A/canine/Florida/20170606_2/2017
MH266313|A/canine/Illinois/A37/2017
MK690072|A/Canine/USA/007780/2018
MH266369|A/canine/Ohio/A82/2017
MH266377|A/canine/Ohio/A91/2017
MH266337|A/canine/Kentucky/174490/2017
MH266361|A/canine/Ohio/174509/2017
MK690056|A/Canine/USA/000915/2018
MH266385|A/canine/Ohio/A97/2017
MH266329|A/canine/Kentucky/128656/2017
MH266321|A/canine/Indiana/130636/2017
MH266345|A/canine/Minnesota/139684/2017
MH266393|A/canine/South_Carolina/132956/2017
MK212420|A/canine/Shanghai/20170721_36/2017
MK212436|A/canine/Shanghai/20170622_7/2017
MK212428|A/canine/Shanghai/20170713_18/2017
MK212444|A/canine/Nanjing/20170704_53/2017
MK212484|A/canine/Nanjing/20170328_7/2017
MK212452|A/canine/Nanjing/20170620_47/2017
MK212476|A/canine/Nanjing/20170419_12/2017
MK212460|A/canine/Nanjing/20170607_40/2017
MK212468|A/canine/Nanjing/20170515_25/2017
MK212500|A/canine/Beijing/20161228_9/2016
MK212508|A/canine/Beijing/20161216_8/2016
KT002539|A/canine/Illinois/12191/2015
KX570976|A/canine/Illinois/2883703/2015
MF173168|A/canine/Indiana/132276_16_1_2/2016
MF173233|A/canine/Illinois/091375_16_1_1/2016
MF173362|A/canine/Indiana/17031_16_2/2016
MF173309|A/canine/Illinois/145135_16_3_1/2016
MF173172|A/canine/Indiana/1177_17_1/2017
MG982726|A/canine/Illinois/M17_05782_7_1/2017
MG982768|A/canine/Illinois/M17_05782_1_1/2017
MG982695|A/canine/Illinois/M17_05782_11_1/2017
MG982790|A/canine/Illinois/M17_05782_4_1/2017
MG982822|A/canine/Pennsylvania/20170411_5/2017
MG982839|A/canine/Illinois/M17_05782_8_1/2017
MG982717|A/canine/Illinois/M16_25374_14_1/2016
MG982702|A/canine/Illinois/M16_16391_2_1/2016
MF173245|A/canine/Illinois/20170110_1A4/2016
MG982722|A/canine/Illinois/M16_30480_3_1/2016
MG982739|A/canine/Illinois/20170110_1G4/2016|
MG982777|A/canine/Illinois/20170110_1G12/2016
MF173247|A/canine/Indiana/093165_16_1_1/2016
MG982760|A/canine/Illinois/20170110_2G1/2016
MG982877|A/canine/Illinois/20170110_2F1/2016
MG982884|A/canine/Illinois/187427_16_7/2016
KX571017|A/canine/Illinois/0843093/2015
KX571055|A/canine/Illinois/1619144/2015
KX570989|A/canine/Indiana/96198/2015
KX570995|A/canine/Wisconsin/19137/2016
MG982883|A/canine/Illinois/M16_25374_19_1/2016
MF173151|A/canine/Illinois/077753/2016
MF173381|A/canine/Illinois/077752/2016
KX571054|A/canine/Texas/343907/2015
MF173227|A/canine/Indiana/003018/2016
MF173342|A/canine/Illinois/278597/2016
MF173270-A/canine/Illinois/328292/2015
MF173281|A/canine/Florida/269770/2015
MF173280|A/canine/Texas/2100186/2015
MF173290|A/canine/Illinois/I0182389/2015
MF173182|A/canine/Illinois/41915/2015
MF173330|A/canine/Illinois/283066/2015
KX570980|A/canine/Georgia/95391/2015
KX571051|A/canine/Georgia/104940/2015
KX571020|A/canine/North_Carolina/109904/2015
MF173259|A/canine/North_Carolina/103297/2015
MF173283|A/canine/Georgia/101875/2015
MF173303|A/canine/Pennsylvania/0261885/2015
MF173400|A/canine/Indiana/2093681/2015
KX571045|A/canine/Korea/0589318/2015
MF173346|A/canine/South_Korea/0173915/2015
KR154332|A/canine/Korea/DG1/2014
MH105838|A/canine/Korea/AS_05/2012
MH105840|A/canine/Korea/AS_08/2012
MH105841|A/canine/Korea/AS_09/2012
MH105836|A/canine/Korea/AS_03/2012
MH105837|A/canine/Korea/AS_04/2012
MH105839|A/canine/Korea/AS_06/2012
MH105842|A/canine/Korea/AS_11/2013
MH105844|A/canine/Korea/AS_15/2013
MH105843|A/canine/Korea/AS_14/2013
JX679528|A/canine/Korea/KRIBB01/2011
JN247622|A/canine/Jiangsu/06/2010
JX414245|A/canine/Guangdong/05/2011
KJ917348|A/canine/Guangdong/01/2013
KJ917356|A/canine/Guangdong/02/2013
KJ917366|A/canine/Guangdong/03/2013
KJ917385|A/canine/Guangdong/04/2013
KJ917374|A/canine/Guangdong/01/2014
KJ917382|A/canine/Guangdong/02/2014
JF714154|A/canine/Zhejiang/1/2010
JN247614|A/canine/Jiangsu/05/2010
JN247606|A/canine/Jiangsu/04/2010
KF042263|A/canine/Liaoning/27/2012
KF042271|A/canine/Liaoning/H6/2012
KF826950|A/canine/Guangdong/12/2012
KF826958|A/canine/Guangdong/23/2012
MG309803|A/canine/China/LG1/2015
MG309811|A/canine/China/LG2/2015
MG386677|A/canine/China/JLM1/2015
MG386685|A/canine/China/JLM2/2015
MK212532|A/canine/Beijing/20131204_349/2013
MK212524|A/canine/Beijing/20131207_365/2013
MK212540|A/canine/Beijing/20131203_342/2013
MK212548|A/canine/Beijing/20131203_341/2013
MK212820|A/canine/Beijing/20140110_2/2014
MK212556|A/canine/Beijing/20131112_279/2013
MK212828|A/canine/Beijing/20140103_90/2014
MK212492|A/canine/Beijing/20121230_62/2012
MK212516|A/canine/Beijing/20121215_34/2012
KF042279|A/canine/Heilongjiang/L1/2013
JN247590|A/canine/Jiangsu/02/2010
JN247598|A/canine/Jiangsu/03/2010
JN247582|A/canine/Jiangsu/01/2009
JX195351|A/canine/Guangdong/2/2011
JX195359|A/canine/Guangdong/3/2011
GU433370|A/canine/Guangdong/1/2007
GU433355|A/canine/Guangdong/2/2006
GU433346|A/canine/Guangdong/1/2006
2005
2007.5
2010
2012.5
2015
2017.5
2020

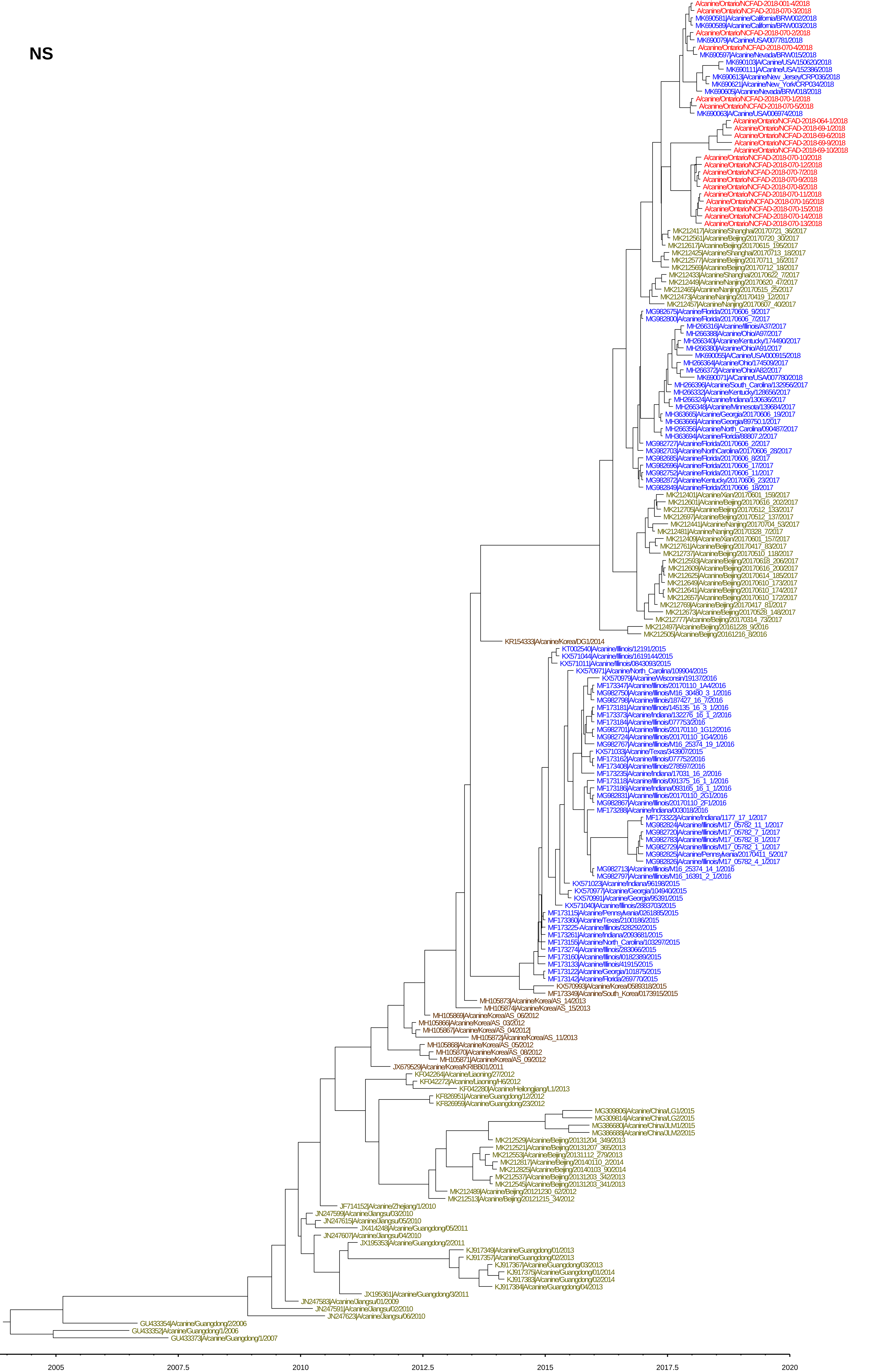
NS
A/canine/Ontario/NCFAD-2018-001-4/2018
A/canine/Ontario/NCFAD-2018-070-3/2018
MK690581|A/canine/California/BRW002/2018
MK690589|A/canine/California/BRW003/2018
A/canine/Ontario/NCFAD-2018-070-2/2018
MK690079|A/Canine/USA/007781/2018
A/canine/Ontario/NCFAD-2018-070-4/2018
MK690597|A/canine/Nevada/BRW015/2018
MK690103|A/Canine/USA/150620/2018
MK690111|A/Canine/USA/152386/2018
MK690613|A/canine/New_Jersey/CRP036/2018
MK690621|A/canine/New_York/CRP034/2018
MK690605|A/canine/Nevada/BRW018/2018
A/canine/Ontario/NCFAD-2018-070-1/2018
A/canine/Ontario/NCFAD-2018-070-5/2018
MK690063|A/Canine/USA/006974/2018
A/canine/Ontario/NCFAD-2018-064-1/2018
A/canine/Ontario/NCFAD-2018-69-1/2018
A/canine/Ontario/NCFAD-2018-69-6/2018
A/canine/Ontario/NCFAD-2018-69-9/2018
A/canine/Ontario/NCFAD-2018-69-10/2018
A/canine/Ontario/NCFAD-2018-070-10/2018
A/canine/Ontario/NCFAD-2018-070-12/2018
A/canine/Ontario/NCFAD-2018-070-7/2018
A/canine/Ontario/NCFAD-2018-070-9/2018
A/canine/Ontario/NCFAD-2018-070-8/2018
A/canine/Ontario/NCFAD-2018-070-11/2018
A/canine/Ontario/NCFAD-2018-070-16/2018
A/canine/Ontario/NCFAD-2018-070-15/2018
A/canine/Ontario/NCFAD-2018-070-14/2018
A/canine/Ontario/NCFAD-2018-070-13/2018
MK212417|A/canine/Shanghai/20170721_36/2017
MK212561|A/canine/Beijing/20170720_30/2017
MK212617|A/canine/Beijing/20170615_195/2017
MK212425|A/canine/Shanghai/20170713_18/2017
MK212577|A/canine/Beijing/20170711_16/2017
MK212569|A/canine/Beijing/20170712_18/2017
MK212433|A/canine/Shanghai/20170622_7/2017
MK212449|A/canine/Nanjing/20170620_47/2017
MK212465|A/canine/Nanjing/20170515_25/2017
MK212473|A/canine/Nanjing/20170419_12/2017
MK212457|A/canine/Nanjing/20170607_40/2017
MG982675|A/canine/Florida/20170606_9/2017
MG982800|A/canine/Florida/20170606_7/2017
MH266316|A/canine/Illinois/A37/2017
MH266388|A/canine/Ohio/A97/2017
MH266340|A/canine/Kentucky/174490/2017
MH266380|A/canine/Ohio/A91/2017
MK690055|A/Canine/USA/000915/2018
MH266364|A/canine/Ohio/174509/2017
MH266372|A/canine/Ohio/A82/2017
MK690071|A/Canine/USA/007780/2018
MH266396|A/canine/South_Carolina/132956/2017
MH266332|A/canine/Kentucky/128656/2017
MH266324|A/canine/Indiana/130636/2017
MH266348|A/canine/Minnesota/139684/2017
MH363665|A/canine/Georgia/20170606_19/2017
MH363666|A/canine/Georgia/89750.1/2017
MH266356|A/canine/North_Carolina/090487/2017
MH363694|A/canine/Florida/88807.2/2017
MG982727|A/canine/Florida/20170606_2/2017
MG982703|A/canine/NorthCarolina/20170606_28/2017
MG982685|A/canine/Florida/20170606_8/2017
MG982696|A/canine/Florida/20170606_17/2017
MG982752|A/canine/Florida/20170606_11/2017
MG982872|A/canine/Kentucky/20170606_23/2017
MG982849|A/canine/Florida/20170606_18/2017
MK212401|A/canine/Xian/20170601_159/2017
MK212601|A/canine/Beijing/20170616_202/2017
MK212705|A/canine/Beijing/20170512_133/2017
MK212697|A/canine/Beijing/20170512_137/2017
MK212441|A/canine/Nanjing/20170704_53/2017
MK212481|A/canine/Nanjing/20170328_7/2017
MK212409|A/canine/Xian/20170601_157/2017
MK212761|A/canine/Beijing/20170417_83/2017
MK212737|A/canine/Beijing/20170510_118/2017
MK212593|A/canine/Beijing/20170618_206/2017
MK212609|A/canine/Beijing/20170616_200/2017
MK212625|A/canine/Beijing/20170614_185/2017
MK212649|A/canine/Beijing/20170610_173/2017
MK212641|A/canine/Beijing/20170610_174/2017
MK212657|A/canine/Beijing/20170610_172/2017
MK212769|A/canine/Beijing/20170417_81/2017
MK212673|A/canine/Beijing/20170528_148/2017
MK212777|A/canine/Beijing/20170314_73/2017
MK212497|A/canine/Beijing/20161228_9/2016
MK212505|A/canine/Beijing/20161216_8/2016
KR154333|A/canine/Korea/DG1/2014
KT002540|A/canine/Illinois/12191/2015
KX571044|A/canine/Illinois/1619144/2015
KX571011|A/canine/Illinois/0843093/2015
KX570971|A/canine/North_Carolina/109904/2015
KX570979|A/canine/Wisconsin/19137/2016
MF173347|A/canine/Illinois/20170110_1A4/2016
MG982750|A/canine/Illinois/M16_30480_3_1/2016
MG982798|A/canine/Illinois/187427_16_7/2016
MF173181|A/canine/Illinois/145135_16_3_1/2016
MF173373|A/canine/Indiana/132276_16_1_2/2016
MF173184|A/canine/Illinois/077753/2016
MG982701|A/canine/Illinois/20170110_1G12/2016
MG982724|A/canine/Illinois/20170110_1G4/2016
MG982767|A/canine/Illinois/M16_25374_19_1/2016
KX571033|A/canine/Texas/343907/2015
MF173162|A/canine/Illinois/077752/2016
MF173408|A/canine/Illinois/278597/2016
MF173235|A/canine/Indiana/17031_16_2/2016
MF173118|A/canine/Illinois/091375_16_1_1/2016
MF173186|A/canine/Indiana/093165_16_1_1/2016
MG982831|A/canine/Illinois/20170110_2G1/2016
MG982867|A/canine/Illinois/20170110_2F1/2016
MF173288|A/canine/Indiana/003018/2016
MF173322|A/canine/Indiana/1177_17_1/2017
MG982824|A/canine/Illinois/M17_05782_11_1/2017
MG982720|A/canine/Illinois/M17_05782_7_1/2017
MG982783|A/canine/Illinois/M17_05782_8_1/2017
MG982729|A/canine/Illinois/M17_05782_1_1/2017
MG982825|A/canine/Pennsylvania/20170411_5/2017
MG982826|A/canine/Illinois/M17_05782_4_1/2017
MG982713|A/canine/Illinois/M16_25374_14_1/2016
MG982797|A/canine/Illinois/M16_16391_2_1/2016
KX571023|A/canine/Indiana/96198/2015
KX570977|A/canine/Georgia/104940/2015
KX570991|A/canine/Georgia/95391/2015
KX571040|A/canine/Illinois/2883703/2015
MF173115|A/canine/Pennsylvania/0261885/2015
MF173360|A/canine/Texas/2100186/2015
MF173225-A/canine/Illinois/328292/2015
MF173261|A/canine/Indiana/2093681/2015
MF173155|A/canine/North_Carolina/103297/2015
MF173274|A/canine/Illinois/283066/2015
MF173160|A/canine/Illinois/0182389/2015
MF173133|A/canine/Illinois/41915/2015
MF173122|A/canine/Georgia/101875/2015
MF173142|A/canine/Florida/269770/2015
KX570993|A/canine/Korea/0589318/2015
MF173349|A/canine/South_Korea/0173915/2015
MH105873|A/canine/Korea/AS_14/2013
MH105874|A/canine/Korea/AS_15/2013
MH105869|A/canine/Korea/AS_06/2012
MH105866|A/canine/Korea/AS_03/2012
MH105867|A/canine/Korea/AS_04/2012|
MH105872|A/canine/Korea/AS_11/2013
MH105868|A/canine/Korea/AS_05/2012
MH105870|A/canine/Korea/AS_08/2012
MH105871|A/canine/Korea/AS_09/2012
JX679529|A/canine/Korea/KRIBB01/2011
KF042264|A/canine/Liaoning/27/2012
KF042272|A/canine/Liaoning/H6/2012
KF042280|A/canine/Heilongjiang/L1/2013
KF826951|A/canine/Guangdong/12/2012
KF826959|A/canine/Guangdong/23/2012
MG309806|A/canine/China/LG1/2015
MG309814|A/canine/China/LG2/2015
MG386680|A/canine/China/JLM1/2015
MG386688|A/canine/China/JLM2/2015
MK212529|A/canine/Beijing/20131204_349/2013
MK212521|A/canine/Beijing/20131207_365/2013
MK212553|A/canine/Beijing/20131112_279/2013
MK212817|A/canine/Beijing/20140110_2/2014
MK212825|A/canine/Beijing/20140103_90/2014
MK212537|A/canine/Beijing/20131203_342/2013
MK212545|A/canine/Beijing/20131203_341/2013
MK212489|A/canine/Beijing/20121230_62/2012
MK212513|A/canine/Beijing/20121215_34/2012
JF714152|A/canine/Zhejiang/1/2010
JN247599|A/canine/Jiangsu/03/2010
JN247615|A/canine/Jiangsu/05/2010
JX414248|A/canine/Guangdong/05/2011
JN247607|A/canine/Jiangsu/04/2010
JX195353|A/canine/Guangdong/2/2011
KJ917349|A/canine/Guangdong/01/2013
KJ917357|A/canine/Guangdong/02/2013
KJ917367|A/canine/Guangdong/03/2013
KJ917375|A/canine/Guangdong/01/2014
KJ917383|A/canine/Guangdong/02/2014
KJ917384|A/canine/Guangdong/04/2013
JX195361|A/canine/Guangdong/3/2011
JN247583|A/canine/Jiangsu/01/2009
JN247591|A/canine/Jiangsu/02/2010
JN247623|A/canine/Jiangsu/06/2010
GU433354|A/canine/Guangdong/2/2006
GU433352|A/canine/Guangdong/1/2006
GU433373|A/canine/Guangdong/1/2007
2005
2007.5
2010
2012.5
2015
2017.5
2020
